# High tumor expression of *CTLA4* identifies lymph node-negative basal-like breast cancer patients with excellent prognosis

**DOI:** 10.1038/s43856-025-00865-z

**Published:** 2025-06-16

**Authors:** Andreas Hagen Røssevold, Xavier Tekpli, Ole Christian Lingjærde, Hege G. Russnes, Johan Vallon-Christersson, Elin Borgen, Jon Lømo, Øystein Garred, Esmaeil Dorraji, Vessela N. Kristensen, Bjørn Naume, Jon Amund Kyte

**Affiliations:** 1 Department of Clinical Cancer Research, Department of Oncology, Oslo University Hospital, Oslo, Norway; 2 Department of Cancer Immunology, Institute for Cancer Research, Oslo University Hospital, Oslo, Norway; 3 Institute of Clinical Medicine, Faculty of Medicine, University of Oslo, Oslo, Norway; 4 Department of Medical Genetics, Clinic for Laboratory Medicine, Oslo University Hospital, Oslo, Norway; 5 Department of Cancer Genetics, Institute for Cancer Research, Oslo University Hospital, Oslo, Norway; 6 Center for Bioinformatics, Department of Informatics, University of Oslo, Oslo, Norway; 7 Department of Pathology, Oslo University Hospital, Oslo, Norway; 8 Division of Oncology, Department of Clinical Sciences Lund, Lund University, Lund, Sweden; 9 Department of Oncology, Oslo University Hospital, Oslo, Norway; 10 Faculty of Health Sciences, Oslo Metropolitan University, Oslo, Norway

**Keywords:** Breast cancer, Cancer microenvironment, Tumour immunology

## Abstract

**Background:**

Tumor immune cell infiltration is a favorable prognostic factor in triple-negative breast cancer. Most triple-negative tumors belong to the aggressive basal-like subtype. We hypothesized that immune gene expression may identify low-risk patients for whom adjuvant chemotherapy can be de-escalated.

**Methods:**

The expression of 753 immune-related genes was analyzed in tumor biopsies from 45 patients with basal-like disease and no lymph node metastases (Oslo1 cohort) and evaluated for prognostic value. Findings were validated in two independent cohorts. Oslo1 biopsies were also analyzed for tumor-infiltrating lymphocytes (TIL) and tertiary lymphoid structures (TLS).

**Results:**

Here we show that a high expression of *CTLA4* (above 63^rd^ percentile) is associated with an excellent prognosis in the Oslo1 cohort. None of the patients in the *CTLA4*^high^ group suffered disease recurrence (median follow-up 7.4 years) or breast cancer-related death (median follow-up 17.7 years). Analysis of the SCAN-B (n = 233; 97% without distant recurrence in CTLA4^high^ group) and METABRIC cohorts (n = 155; 93% disease-specific survival in CTLA4^high^ group) validates this finding, which also applies to patients who did not receive chemotherapy. *CTLA4* expression correlates with TIL score and TLS levels (Oslo1 cohort), but no TIL^low^/*CTLA4*^high^ patients died from breast cancer, suggesting that the *CTLA4* readout identifies low-risk patients not captured by TIL assessment.

**Conclusions:**

A high primary tumor expression of *CTLA4* identifies patients with an excellent prognosis, for whom standard chemotherapy may be de-escalated or omitted.

## Plain language summary

Most breast cancer patients are cured after the initial surgery and radiotherapy. However, basal-like breast cancer, which makes up about 15% of cases, carries a high risk of early recurrence and death. Nearly all patients with basal-like breast cancer therefore receive additional treatment before and after surgery, including chemotherapy and immunotherapy. This treatment reduces the risk of recurrence but causes considerable short- and long-term side effects. In the current study, we found that patients with high levels of the immune-related gene *CTLA4* in tumor had an excellent prognosis, likely because the immune system aids in fighting the cancer. Our findings suggest that the additional treatments given to some patients may safely be omitted or reduced, which could improve the quality of life of cancer survivors.

## Abstract (web summary)

Røssevold et al. identify a subset of patients with early-stage basal-like breast cancer with an excellent prognosis, defined by high tumor expression of the immune checkpoint gene CTLA4. These patients are currently considered high-risk and receive extensive systemic treatment, which the authors suggest could safely be de-escalated or omitted.

## Introduction

Breast cancer (BC) is the most common cancer in women, with approximately 2.3 million new cases diagnosed and 680,000 deaths worldwide in 2020^1^. Approximately 15% of patients with newly diagnosed BC have triple-negative disease. Triple-negative breast cancer (TNBC) is defined by the absence or very low expression of the two hormone receptors (HR) estrogen receptor (ER), and progesterone receptor (PR), and the absence of amplification of human epidermal growth factor receptor 2 (HER2). TNBC has an aggressive phenotype with the highest risk of early recurrence. Despite recent therapeutic advances, survival in metastatic TNBC also remains poor^2–5^. To reduce the risk of disease recurrence and death, the majority of patients receive adjuvant or neoadjuvant chemotherapy, with the addition of immunotherapy for selected cases^6–9^. While adjuvant treatments reduce recurrence rates and mortality, they also cause considerable acute and long-term side effects, with negative impact on the quality of life of patients during and after treatment^10^. Historical data demonstrate that a considerable proportion of patients would be cured by locoregional treatment alone^11^. Identifying low-risk patients at the time of diagnosis could allow de-escalation or omission of the chemotherapy regimen.

Several studies have demonstrated that a high number of tumor-infiltrating lymphocytes (TILs) is a positive prognostic factor in TNBC, as well as a positive predictive factor for response to systemic therapies^12–17^. While various degrees of lymphocyte infiltration can be found in all subtypes of BC, high infiltration is most frequently seen in TNBC^18,19^. The International Immuno-Oncology Biomarker Working Group on Breast Cancer has published guidelines for standardizing the evaluation of TILs and demonstrated that it can be performed in a reproducible manner^20,21^. De Jong et al.^22^ reported an excellent survival outcome without (neo-) adjuvant therapy in patients with TIL score ≥75% (21% of patients) in a cohort of younger patients with lymph-node negative TNBC. Despite standardization efforts, interobserver variation in TIL quantification remains a challenge^23^, and studies with different patient selection criteria and treatment regimens have suggested other prognostic cutoff values. In a pooled analysis of nine studies evaluating TILs as a prognostic marker, Loi and colleagues reported excellent outcomes in lymph node-negative patients with TIL score ≥30%, corresponding to the upper quartile^24^. The 2019 St. Gallen International Consensus Guidelines for the primary treatment of early BC recommended that TILs should be routinely reported in pathology reports for TNBC due to their prognostic value^25^. However, as of the most recent 2023 guidelines, the panel does not recommend that TILs should be used to guide treatment decisions, due to insufficient evidence^6^.

Perou and colleagues introduced the intrinsic subtypes of breast cancer in 2000, based on microarray analyses of tumor expression of 8000 genes^26^. The main subtypes, Luminal A, Luminal B, HER2-enriched, and Basal-like, display differences in biology, clinical behavior, and response to treatment^27–29^. Parker and colleagues^30^ showed that reproducible prediction of the intrinsic subtypes can be performed by expression analysis of 50 genes, known as the PAM50 classifier. While tumor gene expression analyses have become widely available only in recent years, routine immunohistochemistry (IHC) evaluation of the expression of estrogen receptor (ER), progesterone receptor (PR), and HER2 has been universally adopted to assess susceptibility to endocrine and HER2-directed treatment. As most basal-like tumors are triple-negative by IHC, triple-negative disease has been used as a surrogate marker for the basal-like subtype, guiding treatment decisions as well as enrollment and stratification in clinical trials. However, 14% of triple-negative tumors are classified as non-basal by PAM50, and tumors of all intrinsic subtypes can be found within the triple-negative category^28^. Central properties of the intrinsic subtypes are retained regardless of receptor status^31,32^. This diversity within TNBC may explain some of the difficulties in identifying robust prognostic and predictive biomarkers. Addressing this challenge, we performed patient selection by intrinsic subtype for the primary analyses in the current study.

While the presence of immune activation in breast tumors and its correlation with treatment outcome have been known for more than a century^17^, this prognostic information has not been generally implemented to guide treatment decisions. Quantification of TILs by histopathological evaluation of hematoxylin and eosin (H&E) slides represents an affordable option. However, the standardization of such methods remains challenging for implementation in the clinical setting. In the current study, we retrospectively analyzed the expression of a curated set of 753 immune-related genes in basal-like tumors from a prospective study of patients with early-stage BC, with available PAM50-based intrinsic subtype information and known clinical and histopathological risk factors^33^. The aim of the study was to investigate whether immune gene expression patterns can identify patients with low risk of disease recurrence and death, who could benefit from de-escalation or omission of adjuvant chemotherapy.

We show that a high tumor expression of the immune checkpoint gene *CTLA4* is associated with an excellent prognosis and high TIL scores in patients with lymph node-negative, basal-like breast cancer in the Oslo1 study. The correlation of *CTLA4* expression with prognosis is also demonstrated in two independent validation cohorts, SCAN-B and METABRIC.

## Methods

### Ethical approval

The present study was approved by the Regional Committee for Medical Research Ethics South-East Norway (EC ID: 2015/1216 and 2015/2453). Informed, written consent was obtained from all patients in the Oslo1 cohort. Participant information from the other cohorts was obtained in a deidentified format from publicly available sources.

### The Oslo1 cohort

The Oslo Micrometastasis Project (the Oslo1 study) has been described previously^33,34^. This was an observational study where 920 patients with early-stage breast cancer were enrolled between 1995 and 1998 and treated according to national recommendations. We have previously determined the intrinsic subtypes (PAM50) for tumor samples from 666 patients in the Oslo1 cohort^33^. Formalin-fixed, paraffin-embedded (FFPE) biopsies from primary tumors were collected in a research biobank. Clinical and histopathological variables and data on disease relapse status were obtained from hospital records, with the last update completed in 2005. Survival status and cause of death were obtained from hospital records and the Norwegian Institute of Public Health’s Cause of Death Registry. Survival follow-up was completed on Dec 31, 2014. PAM50 intrinsic subtypes were determined for tumor samples from 666 patients, using the nCounter Analysis System and the Prosigna® algorithm (NanoString Technologies, Seattle, WA, US).

### RNA expression analysis from FFPE biopsies

**Fig. 1:**
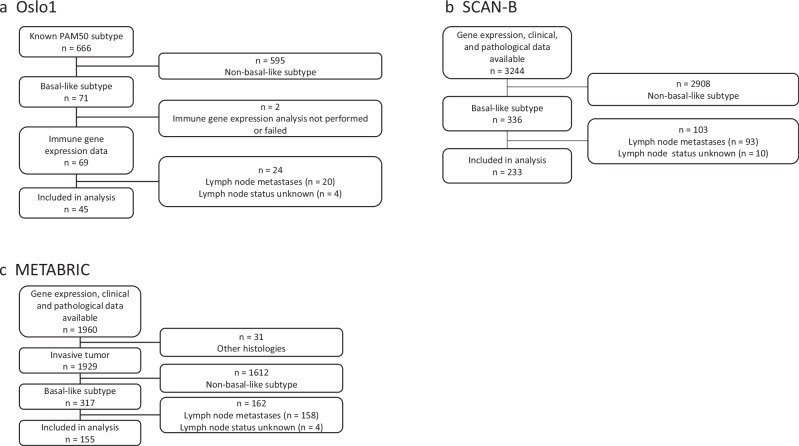
Patient disposition. Selection criteria for patients/samples included in the study for each cohort; Oslo1 (**a**), SCAN-B (**b**), and METABRIC (**c**).

H&E-stained slides from primary tumors from the Oslo1 study were examined by a pathologist in order to identify areas with mainly tumor tissue. RNA was purified from 1 to 5 10 µm slides per tumor using the Roche® High Pure FFPET RNA Isolation Kit, in order to obtain a minimum of 100 ng of RNA from each sample. The RNA expression levels of 760 immune-related genes were analyzed using the PanCancer Immune Profiling Panel on the nCounter platform. This assay covers 730 genes related to immune cells, immune checkpoints, and other components of the adaptive and innate immune response. Our analysis kits also included 30 custom genes. RNA expression data were obtained from 69 of the 71 patients with basal-like disease (Fig. 1a). Samples were run on one of two different panels with some differences in the selection of custom genes. Only the genes present in both panels were included in the analysis, resulting in a dataset with 753 genes. Expression values (counts) were normalized based on 40 housekeeping genes predefined by the manufacturer. After adding 1 to all expression values, the geometric mean G of the 40 housekeeping genes was calculated for each sample, then the arithmetic mean A of all geometric means. The count data for each sample was normalized by multiplying the raw counts by the factor A/G, resulting in G = A for each sample.

### Analysis of H&E slides

The presence and prevalence of TIL, TLS, and germinal centers were evaluated on H&E stained FFPE slides from surgical specimens of whole tumors. Quantification of TILs was done by two experienced breast cancer pathologists, according to the 2014 recommendations of the International TILs Working Group^20^. TLS was defined as aggregates of lymphocytes just outside the tumor–normal tissue border and scored on a scale from 0 to 3. Germinal centers were defined as areas of larger lymphoid cells within the TLS and were scored as absent (0) or present (1). The methods are illustrated in Supplementary Fig. 1.

### Validation datasets

The Sweden Cancerome Analysis Network—Breast (SCAN-B) study is an ongoing prospective population-based study which has enrolled more than 19,000 breast cancer patients since 2010^35–37^. Patients receive treatment according to national and regional guidelines. Gene expression data (Illumina RNAseq) from primary tumors of patients with early-stage breast cancer included in the SCAN-B study^37^ were downloaded from the Gene Expression Omnibus (GEO; National Center for Biotechnology Information, series GSE96058; accession date April 12, 2022). Clinicopathological data for the SCAN-B follow-up cohort were obtained from Staaf et al. 2022 (ref. ^38^), supplemental data. This dataset contains two different classifications of intrinsic tumor subtypes. One corresponds to the original PAM50 schema, whereas the other omits the normal-like centroid when assigning subtype. We performed patient selection based on the latter classification, as it corresponds better to the Prosigna assay used in the Oslo1 dataset.

The METABRIC (Molecular Taxonomy of Breast Cancer International Consortium) dataset includes gene expression analyses (Illumina HT-12 v3) of clinically annotated primary breast cancer specimens from 1971 patients from tumor banks in the UK and Canada^39^. Gene expression analysis was performed by Illumina HT-12 v3. We obtained clinicopathological data from Curtis et al. 2012 (ref. ^39^), while gene expression data were obtained using R package *MetaGxBreast* (version 1.12.0, accession date October 7, 2021).

### Statistics and reproducibility

All statistical analyses were perfomed using R software (version 4.2.2). All *P* values provided are two-sided. Potentially prognostic genes were identified using the Oslo1 dataset as a training set. Genes were filtered by expression level and variance using R package *genefilter (version 1.80.3)*, using a filter requiring that at least 50% of samples have a normalized count >15 (the upper limit of the 95% CI of the mean signal in negative controls) and a coefficient of variance for gene expression across samples of at least 0.6. After filtering, the expression values were log_2_ transformed, and the expression values of each gene were scaled to a mean of 0 and a standard deviation of 1 (Z-score transformation). The Wilcoxon rank-sum test was used in differential expression analyses. Feature selection by the lasso method^40^ was done using R package *glmnet* (version 4.1-6). Lasso was applied to a Cox regression model with the scaled expression levels of the 299 filtered genes as independent variables and DSS as the dependent variable, and to a binomial regression model with the binary outcome of cancer-related death.

In Oslo1, RFI and DRFI were defined as the time from surgery to any recurrence or distant recurrence, respectively. Carcinoma in situ and new primary breast carcinomas were not considered disease recurrence. OS was defined as the time from surgery to death from any cause, and DSS as the time from surgery to death attributed to breast cancer in hospital records or the Norwegian Cause of Death Registry. Patients who did not progress or die during the follow-up period were censored at the last assessment. The corresponding variables for SCAN-B and METABRIC were obtained from the publicly available datasets. Time-to-event variables were computed and compared between groups using the Kaplan-Meier estimator in R packages *survival* (version 3.5-0) and *survminer* (version 0.4.9). Median follow-up times were estimated using the reverse Kaplan-Meier method, using censoring as the event. *P* values for differences in survival between groups were obtained using the log-rank method. The multivariable regression models were created using the Cox proportional hazards model. Intrinsic subtype classification in the METABRIC dataset was done by the method described by Lien et al.^41^, modified by omitting the normal-like centroid, in order to get classifications equivalent to the Prosigna assay. ROC analyses were done using R package *pROC* (version 1.18.0)^42^. Box plots and scatter plots were made using R package *ggplot2* (version 3.4.0), while the correlation plot in Supplementary Fig. 2 was made using *GGally* (version 2.1.2).

## Results

### Patient selection and characteristics

We have previously determined the PAM50 subtypes for tumor samples from 666 patients in the Oslo1 study^33^. Samples from 71 patients were classified as basal-like. Using the nCounter PanCancer Immune Profiling Panel, we assessed the expression of 753 immune-related genes in 69 of these samples. The 45 samples from patients without lymph node metastases were selected for the current study (Fig. 1a). The median follow-up time for this cohort was 7.4 years for recurrence-free interval (RFI) and 17.7 years for overall survival (OS). Patient clinicopathological features are summarized in Table 1.

**Table 1:** Patient characteristics

	OSLO1	SCAN-B	METABRIC
	(N = 45)	(N = 233)	(N = 155)
Age (years)			
Median [Min, Max]	53.6 [31.2, 80.9]	60.0 [30.0, 95.0]	58.9 [31.3, 84.8]
Tumor size (mm)			
Median [Min, Max]	21.0 [10.0, 80.0]	18.0 [0, 90.0]	20.0 [1.00, 100]
Missing	0	4 (1.7%)	2 (1.3%)
Tumor grade			
I	0 (0%)	6 (2.6%)	3 (1.9%)
II	5 (11.1%)	14 (6.0%)	22 (14.2%)
III	40 (88.9%)	199 (85.4%)	127 (81.9%)
Missing	0	14 (6.0%)	3 (1.9%)
Histology			
IDC	37 (82.2%)	200 (85.8%)	126 (81.3%)
ILC	1 (2.2%)	5 (2.1%)	4 (2.6%)
Other	7 (15.6%)	27 (11.6%)	25 (16.1%)
Missing	0 (0%)	1 (0.4%)	0 (0%)
Receptor status			
TNBC	37 (82.2%)	176 (75.5%)	0
HR+/HER2−	5 (11.1%)	34 (14.6%)	0
HR-/HER2+	2 (4.4%)	11 (4.7%)	0
HR+/HER2+	1 (2.2%)	5 (2.1%)	0
Missing	0	7 (3.0%)	155 (100%)^a^
Systemic treatment^b^			
None	27 (60%)	44 (18.9%)	84 (54.2%)
Endocrine therapy only	3 (6.7%)	13 (5.6%)	42 (27.1%)
Chemotherapy only	14 (31.1%)	152 (65.2%)	23 (14.8%)
Chemotherapy + endocrine therapy	1 (2.2%)	22 (9.4%)	6 (3.9%)
Unknown		2 (0.9%)	
Type of surgery			
Breast-conserving surgery	11 (24.4%)		78 (50.3%)
Mastectomy	31 (68.9%)		76 (49.0%)
Unknown	3 (6.7%)	233 (100%)	1 (0.6%)

Distribution of clinical and histopathological variables in the three patient cohorts studied.

*HR* Hormone receptor, *HER2* human epidermal growth factor receptor.

^a^Receptor subtypes could not be determined for METABRIC, as progesterone receptor status was not assessed. The following data were available: ER positive 32 (21%), negative 119 (77%), missing 4 (3%). HER2 positive 10 (6%), negative 57 (37%), missing 88 (57%).

^b^In the Oslo1 study, standard-of-care chemotherapy was given, which at the time was CMF (six cycles of cyclophosphamide 600 mg/m^2^, methotrexate 40 mg/m^2^, and fluorouracil 600 mg/m^2^, given intravenously every 3 weeks). Type of chemotherapy was not specified in the METABRIC or SCAN-B datasets.

Radiation therapy was only specified in the METABRIC dataset, where it was given to 94 patients (61%), predominantly after breast-conserving surgery.

### Identification and selection of prognostic genes

**Fig. 2:**
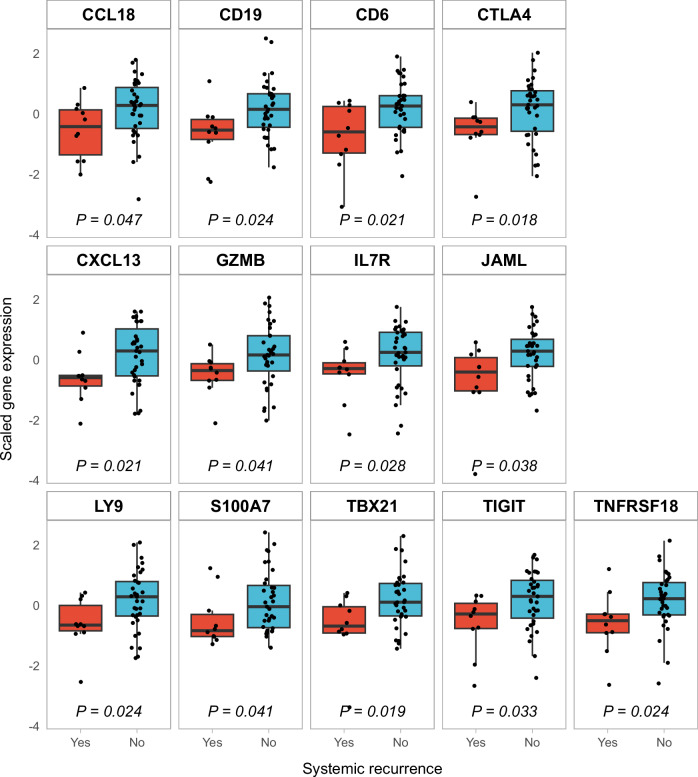
Oslo1: differential gene expression by systemic recurrence. Boxplot of scaled log_2_-transformed normalized gene expression in patients (n = 45) from the Oslo1 cohort with or without subsequent systemic recurrence of breast cancer. *P* values were calculated by Wilcoxon’s rank sum test. In the boxplots, the center line represents the median value, the hinges represent the interquartile range (IQR). Whiskers extend to extreme values, omitting outliers >1.5 × IQR from the box limits.

**Fig. 3:**
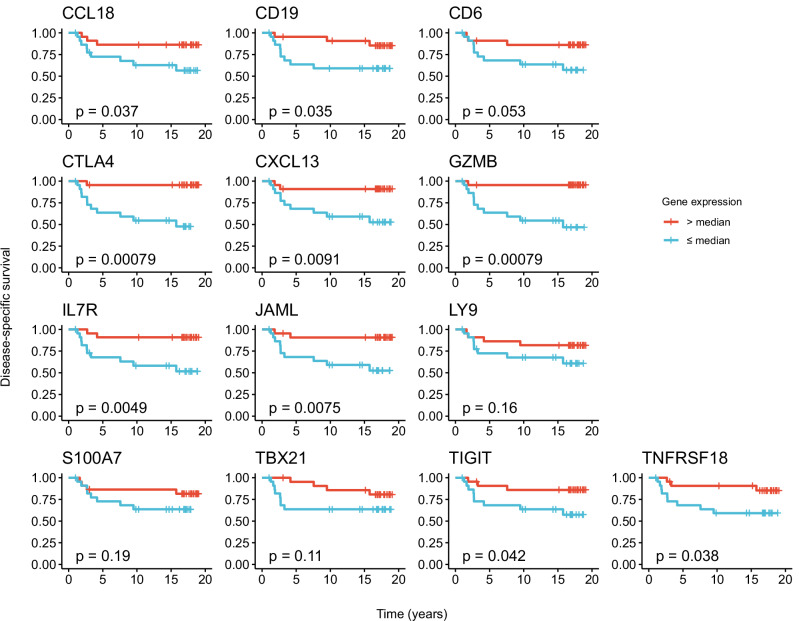
Kaplan-Meier analysis of disease-specific survival in patients with gene expression above and below median. Data from the Oslo1 cohort (n = 45). *P* values were calculated using the log-rank method.

Immune-related genes expressed in <50% of samples or with low variance across samples were removed, resulting in a set of 299 genes. Differential expression analysis identified 13 genes significantly associated with systemic disease recurrence, with a cutoff for significance at *P* < 0.05 without correction for multiple testing. For each of these genes, the expression was higher in the group with no systemic recurrence (Fig. 2). The lowest *P* value was found for Cytotoxic T-lymphocyte-associated protein 4 (*CTLA4*; *P* = 0.018). To investigate whether any of these genes could identify a sizable proportion of patients with a favorable prognosis, we compared the disease-specific survival (DSS) in patients dichotomized by the median expression value for each gene. The DSS was significantly better in the high-expression group for 9 of the 13 genes (Fig. 3). It is worth noting that there was a high degree of correlation between the expression levels of these genes, as shown in Supplementary Fig. 2. The best DSS in the high-expression group and the highest level of significance were found for *CTLA4* and for granzyme B (*GZMB*), which plays an important role in T and NK cell cytotoxicity. For both genes, the 10-year DSS was 95.5% (95% CI 87.1–100%) in the high-expression groups versus 54.5% (95% CI 37.2–79.9%) in the low-expression groups (*P* = 0.00079).

As an alternate feature selection method, we performed Cox regression for DSS with the expression values of the 299 filtered immune genes as predictor variables penalized by the lasso method^40^. This analysis identified high *CTLA4* expression as the only predictor for DSS. We further applied lasso penalization to a binomial regression model with the 299 gene expression values as independent variables and breast cancer-related death as the dependent variable. This analysis also resulted in a model with *CTLA4* expression as the only predictor. As the differential expression analysis and the two lasso regression approaches all suggested a correlation between *CTLA4* expression and DSS, we proceeded to investigate the prognostic value of *CTLA4*.

Performing bivariable Cox regression analyses, *CTLA4* remained a significant predictor for DSS after controlling for age at diagnosis, tumor size, tumor grade, estrogen receptor expression, and Prosigna risk of recurrence (ROR) score (Supplementary Table 1).

### Defining a cutoff value for *CTLA4* expression

**Fig. 4:**
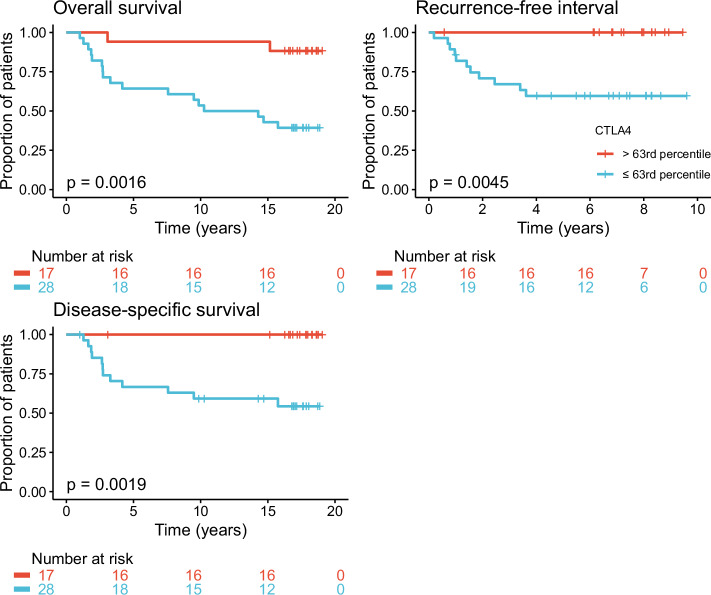
Oslo1: Survival and disease recurrence by *CTLA4* expression. Overall survival, recurrence-free interval, and disease-specific survival in Oslo1 patients with *CTLA4* expression above and below the cutoff at the 63rd percentile.

In order to define a suitable cutoff value for *CTLA4* expression, we performed a ROC analysis with breast cancer-related death as the dependent variable and *CTLA4* expression as the independent variable. The highest unweighted Youden index was found at a threshold value corresponding to the 43rd percentile of *CTLA4* expression, with a sensitivity of 91.7% and a specificity of 75.8% (Supplementary Fig. 3). The unweighted Youden index gives equal weight to false-positive and false-negative values. As misclassifying a high-risk patient as low-risk can have serious consequences, we repeated the analysis using the weighted Youden index. This method takes into account the cost of a false-negative classification compared to a false-positive classification. Setting the relative cost to 3 resulted in a cutoff corresponding to the 63^rd^ percentile of *CTLA4* expression, with a sensitivity of 100% and a specificity of 51.5% for breast cancer-specific death. Figure 4 shows overall survival, recurrence-free interval, and disease-specific survival in patients with *CTLA4* expression above and below the 63^rd^ percentile. None of the patients in the *CTLA4*^high^ group suffered disease recurrence within the follow-up period.

### Validation in other cohorts

The results from the Oslo1 cohort indicated that a high *CTLA4* gene expression in tumors was associated with an excellent prognosis for patients with lymph node-negative basal-like breast cancer. We proceeded to validate this correlation in two unrelated patient cohorts, SCAN-B and METABRIC. The Oslo1 training set and the SCAN-B validation set both arise from population-based, prospective studies of presumably similar populations in Norway and Sweden, respectively. The METABRIC cohort was based on tumor banks in the UK and Canada, and the authors describe the study as population-based^39^. As shown in Table 1, the distribution of central clinicopathological variables is similar across the cohorts. As gene expression data in the three cohorts arise from different technologies, numerical values cannot be compared directly. However, under the assumption that the datasets represent biologically and clinically comparable populations, the distribution of *CTLA4* expression in each cohort would be expected to be similar. Based on the ROC analysis performed in the Oslo1 training set, we chose a threshold for high *CTLA4* expression corresponding to the 63^rd^ percentile in each cohort for validation purposes.

#### SCAN-B

**Fig. 5:**
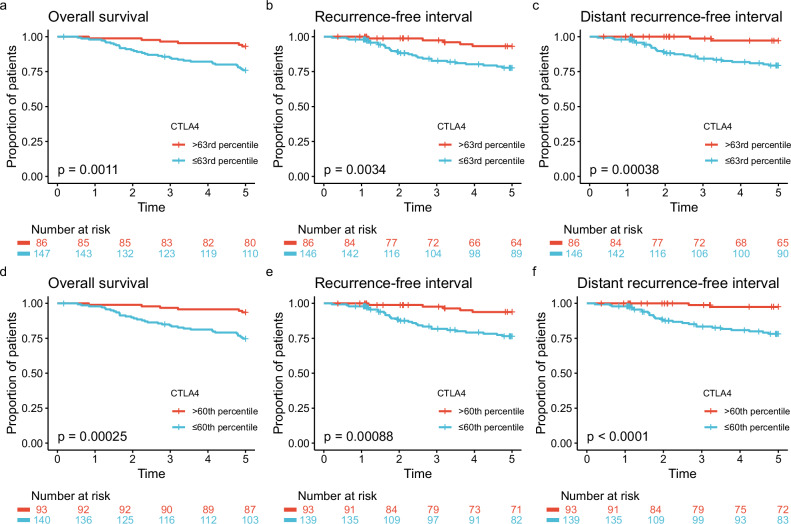
SCAN-B: survival and disease recurrence by *CTLA4* expression. 5-year overall survival, recurrence-free interval, and distant recurrence-free interval in patients with *CTLA4* expression above and below the cutoffs at the 63rd ((**a**–**c**); derived from Oslo1) and the 60th ((**d**–**f**); derived from SCAN-B) percentile in the SCAN-B cohort.

The 233 patients from the SCAN-B study that fulfilled the inclusion criteria were selected as a validation cohort for the current study (Fig. 1b). Patient characteristics are summarized in Table 1. The median follow-up time was 6.8 years for RFI and 8.9 years for OS. We compared 5-year OS, RFI, and distant recurrence-free interval (DRFI) in patients with high versus low expression of *CTLA4*, with a cutoff at the 63rd percentile (Fig. 5a–c). All three outcomes were significantly better in the high-expression group. Only two cases of distant recurrence were recorded in the 86 patients in the high-expression group. Five years after diagnosis, 97.2% had no distant recurrence (95% CI 93.5–100%), and 5-year OS was 93.0% (95% CI 87.8–98.6%). Of the six patients in the *CTLA4*^high^ group who died within five years of diagnosis, three were censored for disease recurrence at the time of death, indicating that a maximum of three of these 86 patients died from breast cancer. To evaluate whether the 63rd percentile cutoff was the best fit for the SCAN-B cohort, we repeated the ROC analysis in the SCAN-B dataset, with distant recurrence as the dependent variable. The weighted Youden index was highest at a threshold corresponding to the 60th percentile (Supplementary Fig. 4). Survival and recurrence outcomes above and below this cutoff in SCAN-B are shown in Fig. 5d–f. We also assessed the effect of using the 60th percentile of *CTLA4* expression to stratify the Oslo1 cohort, as shown in Supplementary Fig. 5.

#### METABRIC

**Fig. 6:**
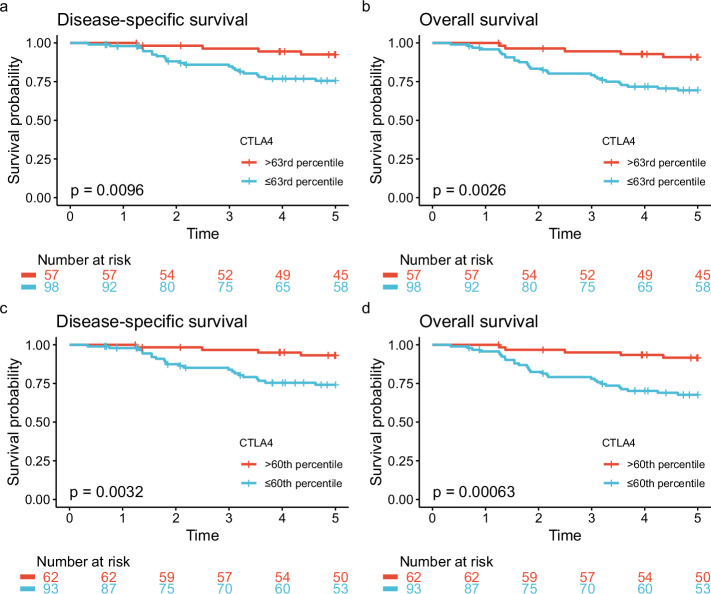
METABRIC: Survival by *CTLA4* expression. Disease-specific and overall survival with *CTLA4* expression above and below the cutoff value at the 63rd (**a**, **b**) and 60th (**c**, **d**) percentile in the METABRIC dataset.

The 155 METABRIC patients that fulfilled the inclusion criteria for the current study were included as a validation cohort (Fig. 1c). Patient characteristics are summarized in Table 1. The median follow-up time for this cohort was 11.9 years for OS and 10.1 years for DSS. With an expression cutoff for *CTLA4* at the 63rd percentile, we found significantly better 5-year OS and DSS in the *CTLA4*^high^ group than in the *CTLA4*^low^ group (Fig. 6a, b). Five-year DSS in the *CTLA4*^high^ group was 92.5% (95% CI 85.8–99.9%), and five-year OS was 90.9% (95% CI 83.5–98.8%). Survival outcomes stratified by the 60^th^ percentile cutoff derived from ROC analysis in the SCAN-B cohort are shown in Fig. 6c, d. The 5-year DSS was 93.2% (95% CI 86.9–99.9%) in the *CTLA4*^high^ group, as defined by the 60th percentile.

### Prognostic value of *CTLA4* expression in the absence of adjuvant chemotherapy

If the prognostic value of *CTLA4* expression is to be used for de-escalation of adjuvant therapy, it is necessary to exclude that high expression merely predicts favorable chemotherapy responses. We performed separate survival analyses for patients treated with and without chemotherapy. The proportion of patients receiving chemotherapy in each cohort is shown in Table 1. In the Oslo1 study, chemotherapy consisted of six cycles of intravenous cyclophosphamide, methotrexate, and fluorouracil (CMF) given every 3 weeks. Chemotherapy regimens were not specified in the validation datasets. Clinicopathological features of patients treated with and without chemotherapy in each of the three cohorts are presented in Supplementary Table 2. In each cohort, patients who did not receive chemotherapy were significantly (*P* < 0.05) older, with the greatest difference in the more recent SCAN-B cohort. Chemotherapy-treated patients had significantly larger tumors in Oslo1. In SCAN-B, patients treated with chemotherapy had significantly higher proportions with high-grade tumors, ductal carcinomas, and triple-negative receptor status.

**Fig. 7:**
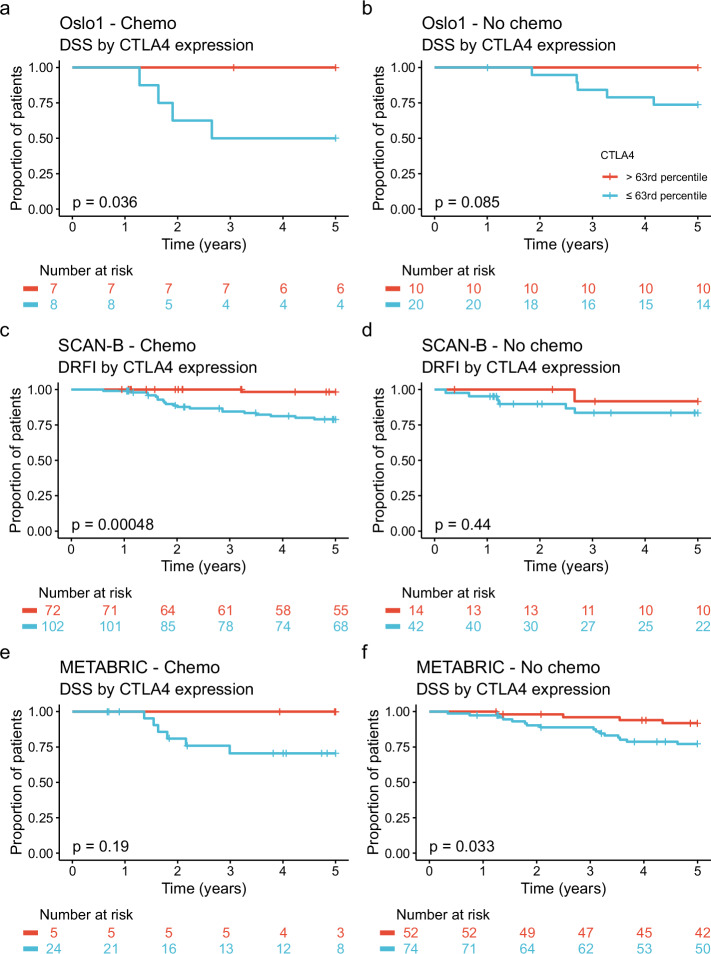
Outcomes in patients with and without (neo-) adjuvant chemotherapy. 5-year outcomes by *CTLA4* expression in patients with (**a**, **c**, **e**) and without (**b**, **d**, **f**) chemotherapy in the Oslo1, SCAN-B, and METABRIC cohorts. The cutoff for high *CTLA4* expression was set at the 63rd percentile within each cohort. Disease-specific survival (DSS) was used as outcome measure for Oslo1 and METABRIC, while distant recurrence-free survival (DRFI) was used for SCAN-B, as cause of death was not available for this cohort.

In all three cohorts, the clinical outcome was better for the *CTLA4*^high^ group, regardless of whether patients received chemotherapy (Fig. 7). The difference was significant (*P* < 0.05) in Oslo1 and SCAN-B for patients treated with chemotherapy and in METABRIC for those treated without. For *CTLA4*^high^ patients who did not receive chemotherapy, 5-year DSS was 100% in Oslo1 and 91.8% (95% CI 84.4–99.8%) in METABRIC, while the proportion with a 5-year DRFI in SCAN-B was 91.7% (95% CI 77.3–100%). For *CTLA4*^high^ patients who did receive chemotherapy, 5-year DSS was 100% in Oslo1 and METABRIC, while 5-year DRFI in SCAN-B was 98.3% (95% CI 95.1–100%).

### *CTLA4* expression and known prognostic factors

The distribution of known prognostic factors in the *CTLA4* high and low groups in the combined cohorts is shown in Supplementary Table 3. The *CTLA4*^high^ group had significantly smaller tumors (mean 20.2 mm vs 23.6 mm, *P* = 0.034). However, improved outcomes in *CTLA4*^high^ tumors were seen both with larger and smaller tumors (Supplementary Figs. 6,7). The *CTLA4*^high^ group also had a significantly higher proportion of patients with grade III tumors, which is a negative prognostic factor. There were no significant differences in age or receptor status between the groups. Data for each cohort are shown in Supplementary Table 4. Patients in the SCAN-B *CTLA4*^high^ group were significantly younger (mean 57 vs 62 years) with a similar trend in Oslo1. They also had significantly smaller tumors (mean 18 vs 22 mm) with a similar trend across all cohorts.

We performed a series of bivariable Cox regression analyses in the two validation cohorts, with DRFI as the outcome measure in SCAN-B and DSS in METABRIC. In both cohorts, *CTLA4* remained a significant predictor after controlling for age, tumor grade, and ER status (Supplementary Tab. 5). When controlling for tumor size, *CTLA4* expression remained significant in SCAN-B (HR 0.63, *P* = 0.011), but not in METABRIC (HR 0.72, *P* = 0.09).

### Correlation between *CTLA4* expression and other measures of tumor inflammation

**Fig. 8:**
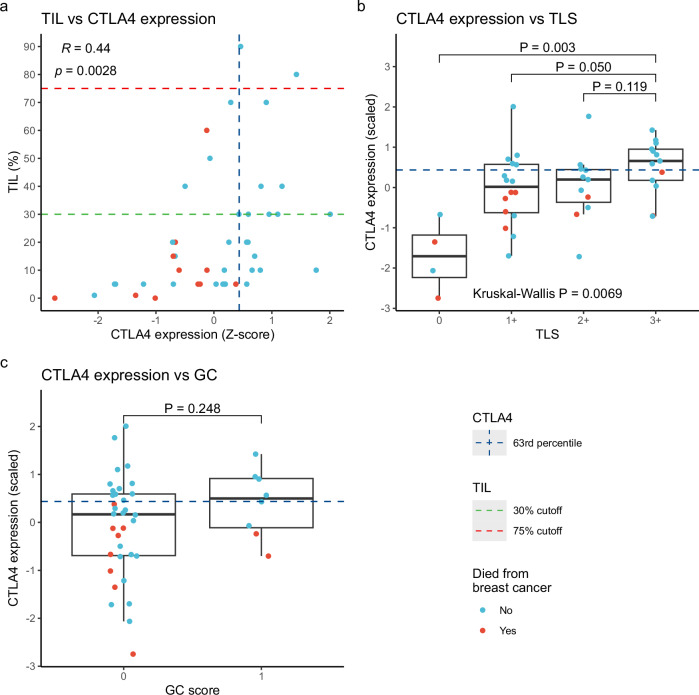
*CTLA4* expression versus TIL, TLS, and GC score. **a** Correlation plot of tumor-infiltrating lymphocytes (TIL) vs *CTLA4*. **b** *CTLA4* expression in samples grouped by prevalence of tertiary lymphoid structures (TLS). **c** *CTLA4* expression in samples grouped by prevalence of germinal centers (GC). Data from the Oslo1 cohort (basal-like N0). All patients with biopsies available for evaluation were included in this analysis. n = 44 for TIL and TLS scores, n = 42 for GC score. Normalized *CTLA4* counts were log_2_ transformed and scaled to a mean of 0 and a standard deviation of 1. TILs were scored according to recommendations by the International TILs Working Group (2014). In the box plots, the center lines represent median values, hinges the IQR, and whiskers the extreme values, omitting outliers extending >1.5 × IQR from the hinges. In boxplots, *P* values were calculated using the Wilcoxon rank-sum test. CTLA4, cytotoxic T-lymphocyte-associated protein 4; *R*, Pearson correlation coefficient.

We further evaluated the correlation between *CTLA4* expression and other markers of tumor inflammation. Quantification of stromal tumor-infiltrating lymphocytes (TIL) was performed according to international guidelines^20^ on H&E-stained slides from the 44 Oslo1 patients for whom biopsies were available for evaluation. While we found a significant correlation between *CTLA4* expression and TILs, a considerable proportion of the tumors with high *CTLA4* expression had low TIL scores (Fig. 8a). None of these *CTLA4*^high^/TIL^low^ patients died from breast cancer. In line with the findings of Loi and colleagues^24,43^, only one of the 14 patients with TIL ≥ 30% died from breast cancer (5-year DSS 93% vs 76% with TIL < 30%, *P* = 0.064). Two patients had TIL scores ≥75%, the prognostic cutoff suggested by Jong et al. ^22^. These patients were both in the *CTLA4*^high^ group, and none of them had disease recurrence or died from breast cancer during the follow-up period.

We evaluated the prevalence of tertiary lymphoid structures (TLS) and germinal centers on the same H&E slides. As shown in Fig. 8b, c, *CTLA4* expression levels were higher in tumors with a higher prevalence of TLS, while no significant difference was observed between tumors with and without germinal centers. Finally, we compared *CTLA4* expression with each of the signatures provided by NanoString based on gene expression data from the PanCancer Immune assay. As shown in Supplementary Fig. 8, a broad range of immune-related signatures were significantly higher in the *CTLA4*^high^ group, including B- and T-cell, cytotoxicity, inflammatory chemokine, interferon gamma, immune checkpoint, and tumor inflammation signatures^44^. None of these signatures included *CTLA4* expression.

### Triple-negative breast cancer

**Fig. 9:**
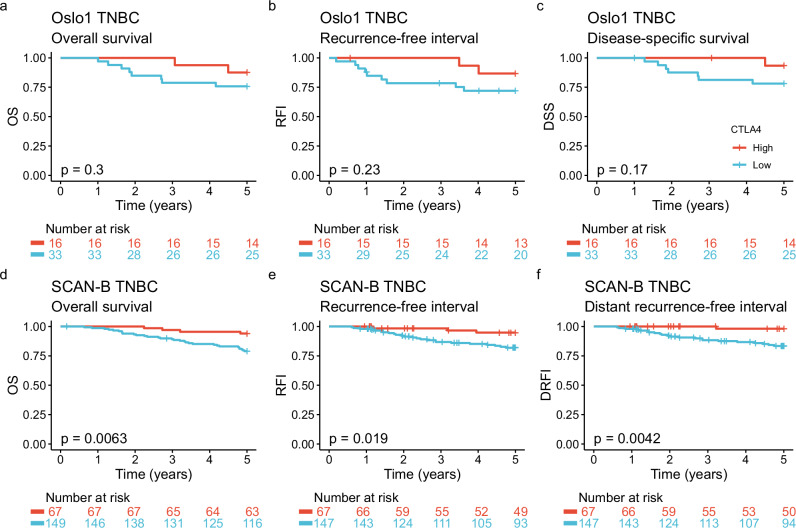
Outcomes by *CTLA4* expression in triple-negative breast cancer. Survival/recurrence rates in patients with high and low *CTLA4* expression and triple-negative breast cancer (regardless of molecular subtype) in Oslo1 (**a**–**c**) and SCAN-B (**d**–**f**). For each cohort, the cutoff for high *CTLA4* expression was set at the 63rd percentile of gene expression in basal-like samples in the same cohort.

To investigate whether our findings could be extended to TNBC in general, we compared outcomes with high and low *CTLA4* expression for patients with lymph node-negative TNBC as defined by IHC, regardless of molecular subtype. This analysis was performed in Oslo1 and SCAN-B, whereas IHC data were not available for the METABRIC cohort. The cutoff for high *CTLA4* gene expression was kept at the 63rd percentile for basal-like samples in each cohort. As shown in Fig. 9, outcomes were numerically favorable for patients with high *CTLA4* expression in both cohorts. In the Oslo1 *CTLA4*^high^ group, 86.7% (95% CI 71.1–100%) were recurrence-free after 5 years, while 5-year DSS was 92.9% (80.3–100%). In SCAN-B, 98.2% (95% CI 94.6–100%) had no distant recurrences after five years, and five-year OS was 93.3% (95% CI 81.5–100%). The improved outcomes with high *CTLA4* were not statistically significant in Oslo1. In SCAN-B, OS, RFI, and DRFI were all significantly better for TNBC patients with a high *CTLA4* expression. Outcomes stratified by the 60th percentile cutoff are shown in Supplementary Fig. 9.

### Development of a single-sample assay for *CTLA4* expression

While a percentile cutoff can be used to categorize each sample in a representative dataset as *CTLA4* high or low, it is not useful for categorizing single samples. If *CTLA4* expression is to be used as a clinical test, a single-sample assay must be developed. One strategy for achieving this is assessing the ratio between *CTLA4* expression and the expression of housekeeping genes, which are consistently expressed across tissues and cell types. The NanoString panel used for gene expression analysis in the Oslo1 cohort contains 40 housekeeping genes. We developed a single-sample score based on this principle. First, the value one was added to the raw count value of all genes in order to eliminate zero counts in the geometric mean calculation. We then calculated the ratio between the raw expression value of *CTLA4* and the geometric mean of the raw expression values of the 40 housekeeping genes for each patient. This ratio was then log_2_ transformed. This method is analogous to the normalization procedure used in the NanoString dataset, thus, there is a linear correlation between the normalized log_2_ transformed *CTLA4* expression used in the above analyses and the single-sample score. This means that the quantile distribution is preserved, and the performance of this score is identical to that of the normalized *CTLA4* expression. The distribution of the single-sample score is shown in Supplementary Fig. 10. The score is roughly normally distributed (Shapiro-Wilks *P* = 0.549). The 63rd percentile corresponds to a score of −0.649.

## Discussion

In this study, we have identified a subgroup of patients with lymph-node negative basal-like early-stage breast cancer with excellent outcomes, characterized by high tumor gene expression of *CTLA4*. Favorable outcomes are seen in patients treated with and without adjuvant chemotherapy. Tumor *CTLA4* expression was positively correlated with the prevalence of TILs and TLS in tumors and with a wide range of immune-related gene expression signatures, including the cytotoxicity, interferon gamma, and tumor inflammation signatures. These results suggest that *CTLA4* expression is a marker for an ongoing anti-tumor immune response. It may seem counterintuitive that *CTLA4*, an inhibitory immune checkpoint, represents a marker for an active immune response. However, *CTLA4* expression is low in naïve T cells, and rapidly increases upon activation in order to maintain homeostasis. The activation of this inhibitory signaling pathway is therefore a sign of immune activation.

It is well known that immune activation is a favorable prognostic factor in basal-like BC. We aimed to advance beyond the identification of prognostic factors to identify a biomarker that may be clinically useful for de-escalation of therapy. This required tuning of the cutoff, so that a group with excellent prognosis could be identified. The ROC analysis in the Oslo1 cohort produced a 63rd percentile cutoff, which was validated in the SCAN-B and METABRIC cohorts. As the Oslo1 cohort was relatively small, we performed a second ROC analysis on SCAN-B data, resulting in a 60th percentile cutoff, which was validated in the Oslo1 and METABRIC cohorts. As would be expected, the 63rd percentile cutoff derived from the Oslo1 dataset performed better in Oslo1, while the 60th percentile cutoff performed better in SCAN-B. In METABRIC, the DSS, OS, and their respective *P* values were slightly better using the 60th percentile. The fact that the two identified cutoff values were close to each other suggests that an appropriate *CTLA4* cutoff for de-escalation strategies is in the range of 60–63% and provides a basis for prospective validation studies. The treatment recommendations changed from the time of the Oslo1 study (1995–1998) to the more recent SCAN-B study (after 2010), with more adjuvant chemotherapy given for TNBC. The fact that the *CTLA4* cutoff is still relatively similar is consistent with the notion that this subgroup has an excellent prognosis regardless of adjuvant therapy. As the cutoff is relative, it will depend on the sampling and analysis protocol, and on the patient population in each cohort. We suggest a method for developing a single-sample test for nCounter data. In the training set, the performance of this score is identical to that of the percentile cutoff. However, as the gene expression data in the three cohorts are derived from different methodologies, verification could not be performed using the datasets presented here. Other methods for deriving a single-sample score must be developed if *CTLA4* status is to be determined by other gene expression technologies.

A correlation between *CTLA4* expression and treatment outcomes in breast cancer has been described previously, but a subgroup with excellent prognosis has not been identified. In a retrospective analysis of the prognostic value of 50 immune checkpoints in BC, Fang and colleagues found that *CTLA4* gene expression was upregulated in breast tumors compared to normal breast tissue, with the highest expression found in TNBC^45^. They found that recurrence-free survival and OS were improved in patients with high expression of several immune genes, including *CTLA4*. Yuan and colleagues also reported improved outcomes in TNBC patients with high *CTLA4* expression, and within the subgroup that also had a high prevalence of intratumoral lymphocytes^46^. In a biomarker study including the expression of 12 immune-related genes in patients from the GeparSixto trial, *CTLA4* was found to be a significant predictor of complete pathological response to neoadjuvant chemotherapy in TNBC^47^. Evaluating CTLA4 expression in BC by IHC, Yu and colleagues found that a high density of CTLA4-positive lymphocytes was an independent predictor of longer OS, while high expression of CTLA4 in tumor cells was a predictor of shorter OS, after controlling for clinicopathological variables including ER, PR, and HER2. Stratified results for intrinsic or receptor-based subtypes were not reported. Interestingly, they found that while CTLA4 was localized to the membranes of lymphocytes, the CTLA4 found in tumor cells appeared to be of the soluble isoform. The two isoforms cannot be distinguished by the *CTLA4* assay used in our study, but selective probes for each of the isoforms are available on the NanoString nCounter platform and could be used in future prospective studies.

The sample size of the Oslo1 cohort used for biomarker discovery was limited. Combined with a high number of variables (753 genes), this leads to a considerable risk of false-positive findings. The use of lasso-penalized regression reduces the risk of model overfitting. Furthermore, the successful validation of the findings in two larger, independent cohorts supports the potential clinical impact of our findings, as does the concordance with previously published data. Our results are, however, based on retrospective analyses. The cohorts studied were subject to different treatment regimens, and different assays were used to identify basal-like tumors. These challenges could be addressed in a prospective verification study.

The EBCTCG meta-analysis of 123 randomized trials indicated that adjuvant anthracycline–taxane combinations reduce breast cancer-related mortality by approximately 1/3, largely independent of tumor characteristics and other known prognostic factors^11^. This means that the potential benefit of treatment mainly depends on the risk at baseline. A 5-year risk of distant recurrence of 10% is a commonly used threshold to balance the risks and benefits of adjuvant chemotherapy^48^. Five-year DRFI for chemotherapy-untreated patients with *CTLA4*^high^ basal-like BC was 100% in the Oslo1 cohort and 93.3% in SCAN-B. This suggests that the risks related to adjuvant chemotherapy may outweigh the small potential benefit for these patients. The risk of recurrence is similar to or lower than that associated with small (pT1a-b) tumors and node-negative disease. These low-risk patients were not included in the pivotal randomized trials comparing chemotherapy to no chemotherapy. However, several retrospective analyses of observational studies have shown similar outcomes with and without adjuvant chemotherapy for these patients^49,50^. Given the similar risk of recurrence, this suggests that patients with node-negative basal-like BC and a high tumor *CTLA4* expression may safely forego adjuvant chemotherapy.

A less radical de-escalation approach would be to use a less intensive chemotherapy regimen, such as an anthracycline and cyclophosphamide without a taxane (AC), or a taxane and cyclophosphamide without an anthracycline (TC). As *CTLA4*^high^ tumors show signs of a high immune activity, a third option might be a de-escalated chemotherapy regimen given in combination with an immune checkpoint inhibitor, or the replacement of chemotherapy with checkpoint inhibitors. These de-escalation strategies may also be relevant in the neoadjuvant setting, where immunotherapy is currently standard of care for a subset of patients^51^. The goal of each approach would be to avoid or reduce the short- and long-term toxicities of the potent but toxic multi-agent chemotherapy regimens currently given to these patients. Such options need to be evaluated in randomized de-escalation studies against standard-of-care treatment. Prospective validation of *CTLA4* as a biomarker could also be done in ongoing studies evaluating other de-escalation strategies.

Assessment of stromal TILs has shown promise as a prognostic biomarker in early-stage TNBC, and efforts have been made to include TILs in staging systems and treatment guidelines^43^. While TILs and *CTLA4* are both markers of immune activation, the presence of TIL^low^/*CTLA4*^high^ and TIL^high^/*CTLA4*^low^ patients (Fig. 8a) suggests that the two biomarkers could be complementary. The excellent clinical outcome in the TIL^low^/*CTLA4*^high^ group suggests that *CTLA4* expression identifies additional patients with a good prognosis. We did not observe a similar favorable outcome for the TIL^high^/*CTLA4*^low^ group, but the numbers are small, and we cannot, based on our data, determine if a combination of the methods may identify a higher number of low-risk patients than each method alone.

With the implementation of multigene assays in clinical decision making for HR-positive, HER2-negative BC, gene expression assays have become more available and cost-efficient. Gene expression analyses, including molecular subtyping, can be performed from standard diagnostic FFPE samples. The OPTIMA Prelim study concluded that such tests could be cost-effective in HR+ node-negative BC^52^. Our findings suggest that with the addition of *CTLA4* expression, this technology could offer a prognostic test also for basal-like BC, with therapeutic consequences.

The TNBC subgroup is biologically heterogeneous and based on a negative definition, characterized by the absence of targets for therapy that were available at the time when this classification was introduced. After the emergence of immunotherapy, TNBC is for practical purposes, divided into PD-L1^positive^ and PD-L1^negative^ disease^5,53^. The effect of trastuzumab deruxtecan on HER2^low^ disease^54^, moreover, is likely to lead to reclassification of TNBC cases. The findings in our study provide further evidence suggesting a need to refine the current TNBC classification and implement markers of immune activation.

In conclusion, the present study identifies high *CTLA4* gene expression as a biomarker for excellent prognosis in patients with primary, lymph node-negative, basal-like breast cancer. A single-gene *CTLA4* score may be combined with other gene expression assays and used for de-escalation of adjuvant and neoadjuvant chemotherapy, thereby preventing side effects and improving the quality of life for cancer survivors. For clinical implementation, there is a need to validate this prospectively.

## Supplementary information

Supplementary Information

Description of Additional Supplementary Materials

Supplementary Data 1

Transparent Peer Review file

## Data Availability

Source data underlying the analyses presented herein are available in Supplementary Data 1. Additional data from the Oslo1 cohort are available from the corresponding author upon reasonable request. Gene expression data from the SCAN-B cohort are available from GEO (accession number GSE96058), and additional clinicopathological data are available in Staaf et al. 2022 (ref. ^38^), supplementary information. Gene expression data from the METABRIC cohort are available from the European Genome-Phenome Archive (EGA; accession number EGAS00000000083). Clinicopathological data are available in Curtis et al. 2012 (ref. ^39^), supplementary information.
