# Supplementary material for: High tumor expression of CTLA4 identifies lymph node-negative basal-like breast cancer patients with excellent prognosis
Source: Commun Med (Lond). 2025 Jun 16;5:234. doi: 10.1038/s43856-025-00865-z (PMC12170890; doi:10.1038/s43856-025-00865-z)
Supplement: Supplementary file 1 — Supplementary Information [file 43856_2025_865_MOESM1_ESM.pdf]

Supplementary Figures

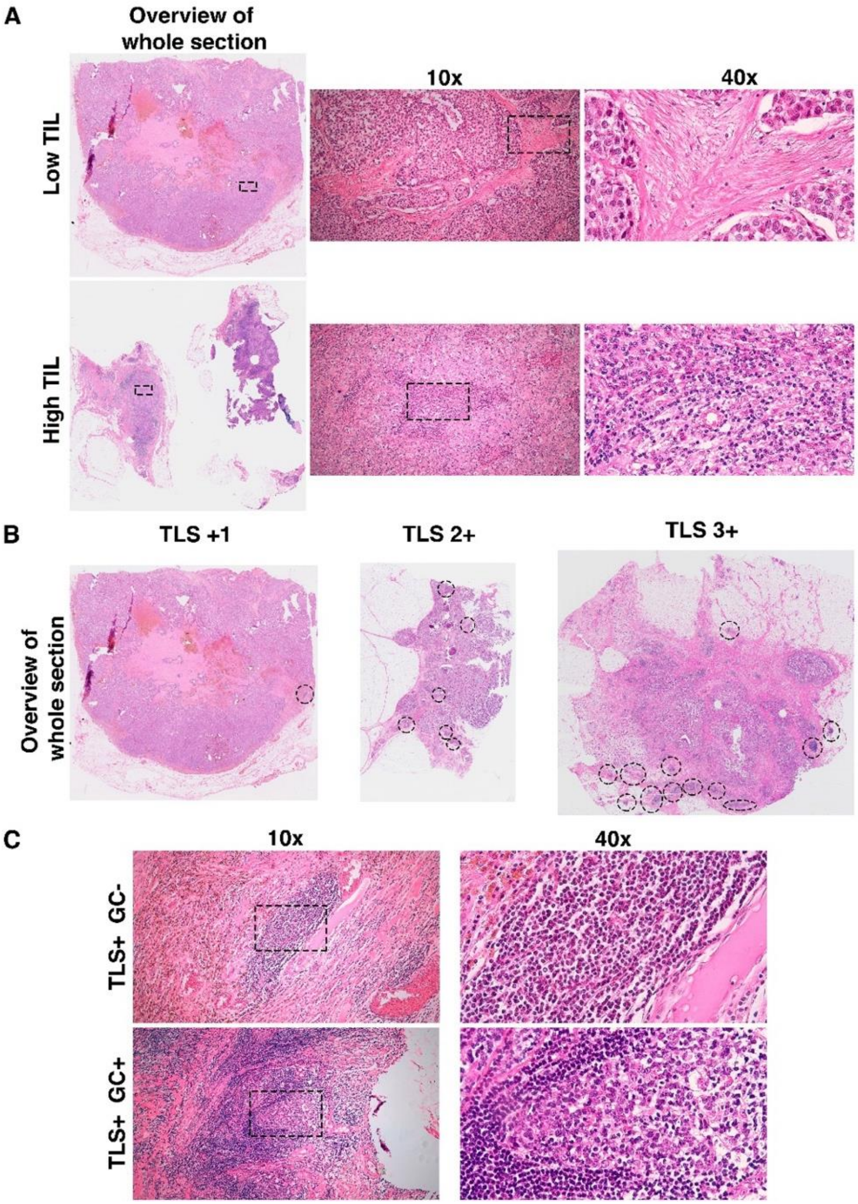

Supplementary Fig. 1 | Evaluation of tumor-infiltrating lymphocytes, tertiary lymphoid structures, and germinal centers on H&E slides

A: Examples of tumors with low (5%) and high (80%) TIL scores.

B: Scoring of tertiary lymphoid structures (TLS) on whole tumor sections.

C: TLS with and without germinal centers, at enhanced magnification.

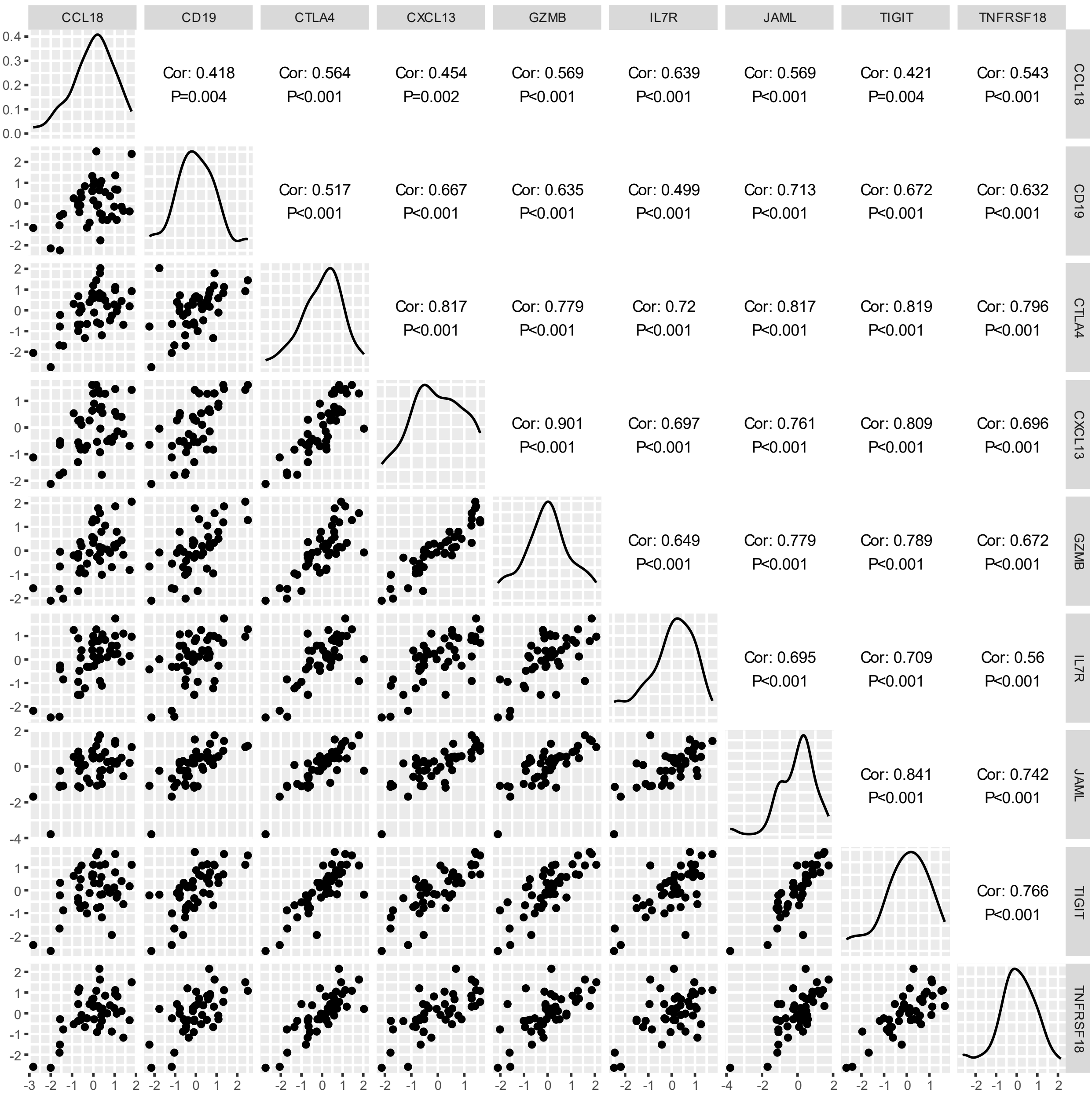

Supplementary Fig. 2 | Gene expression correlation

Pairwise correlations between the scaled expression of the 9 genes that were significantly correlated with disease-specific survival in the Oslo1 cohort.

Cor = Pearson’s correlation coefficient.

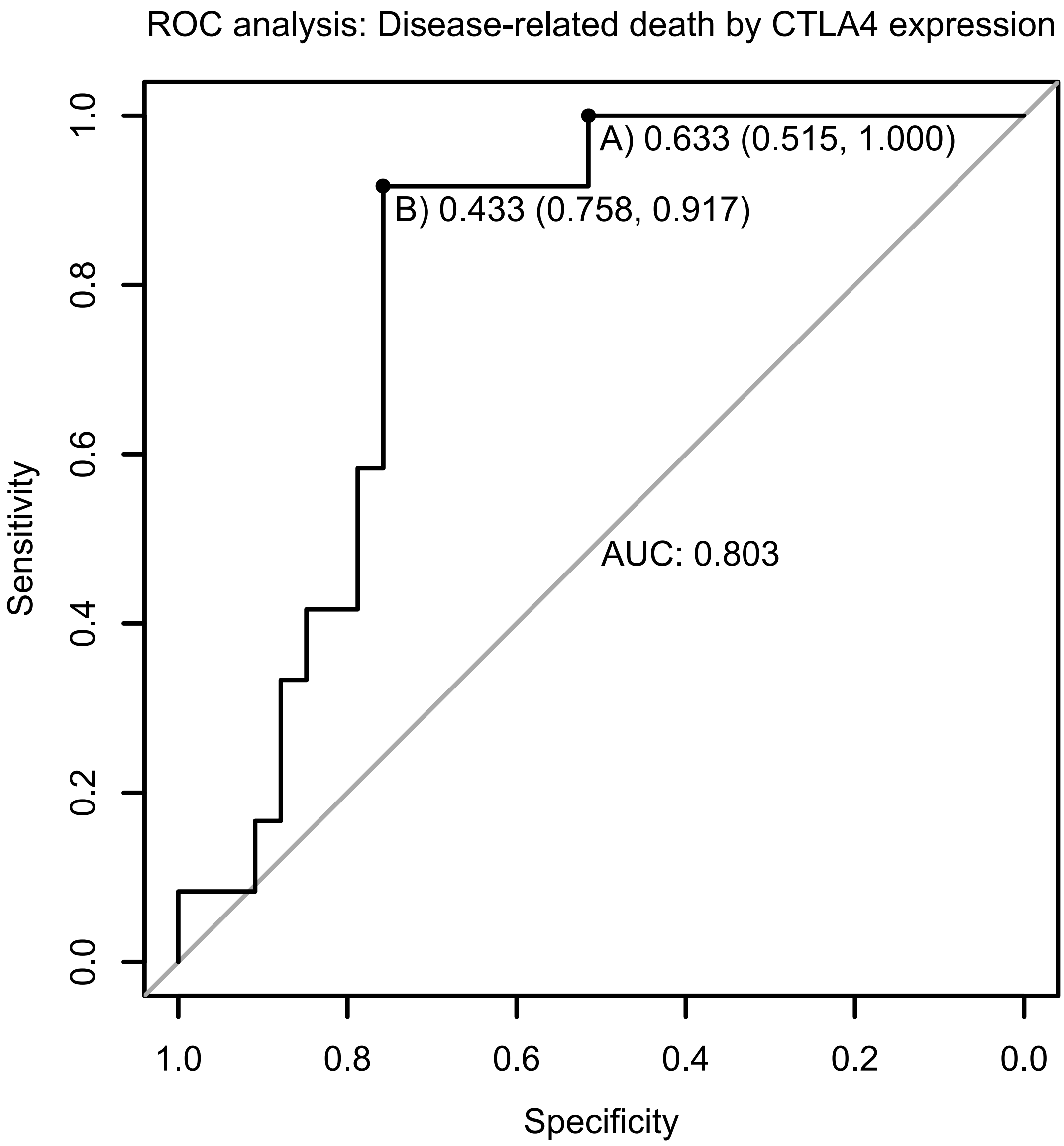

Supplementary Fig. 3 | ROC analysis

ROC analysis was performed with breast cancer-related death as a response variable and *CTLA4* expression (transformed to quantiles) as a predictor variable (n = 45). The best *CTLA4* expression threshold determined by the weighted Youden index corresponds to the 63rd percentile of *CTLA4* expression, with a sensitivity of 1.000 and a specificity of 0.515 for disease-related death (A). With the unweighted Youden index, the best threshold corresponds to the 43rd percentile, with a sensitivity of 0.917 and a specificity of 0.758 for disease-related death (B).

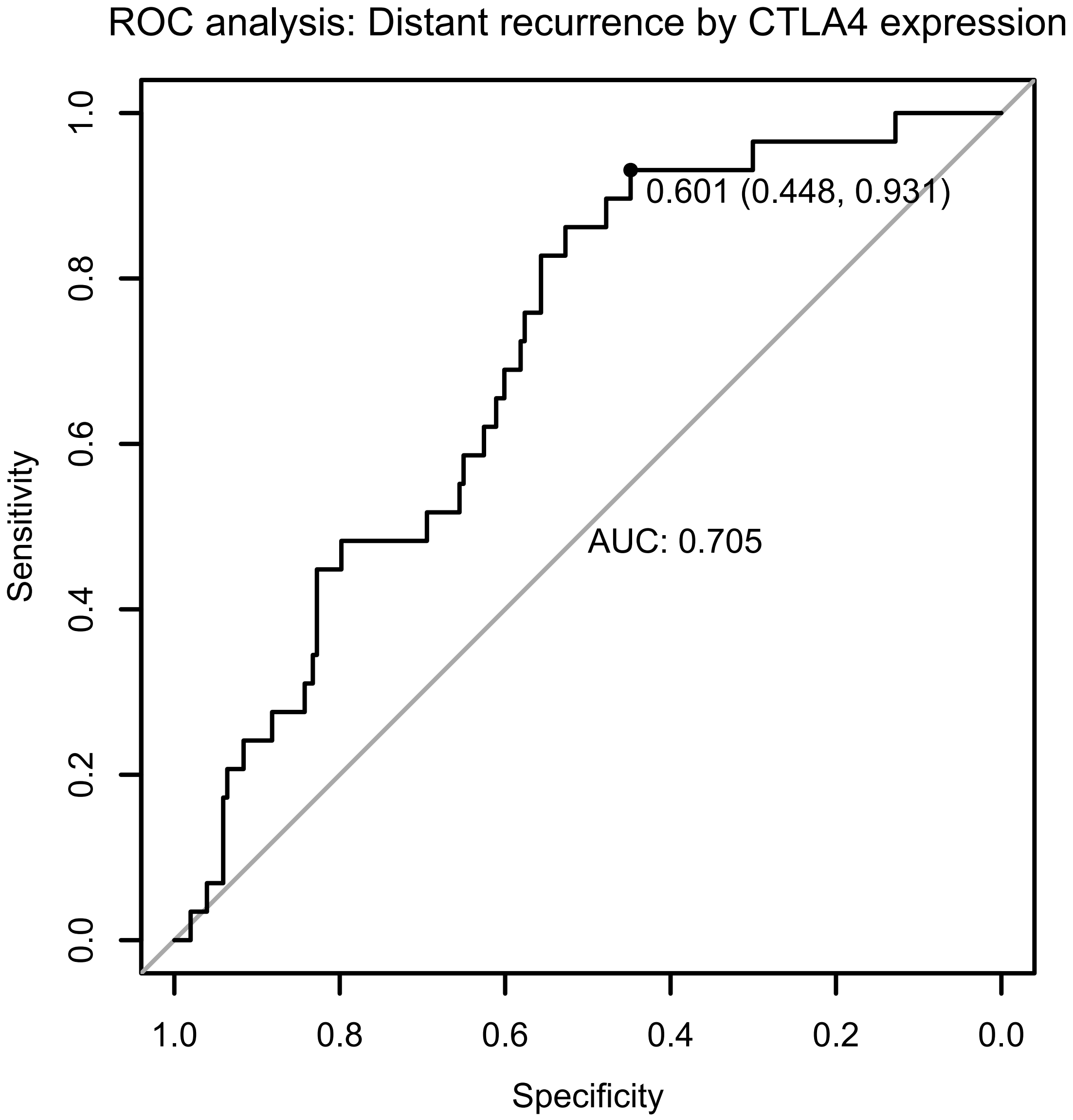

Supplementary Fig. 4 | ROC analysis

ROC analysis of distant disease recurrence by *CTLA4* expression quantiles in the SCAN-B validation cohort (recurrence status available for n = 232). The best threshold at the 60th percentile was determined by the weighted Youden index, with the relative cost of a false negative versus a false positive classification set to 3.

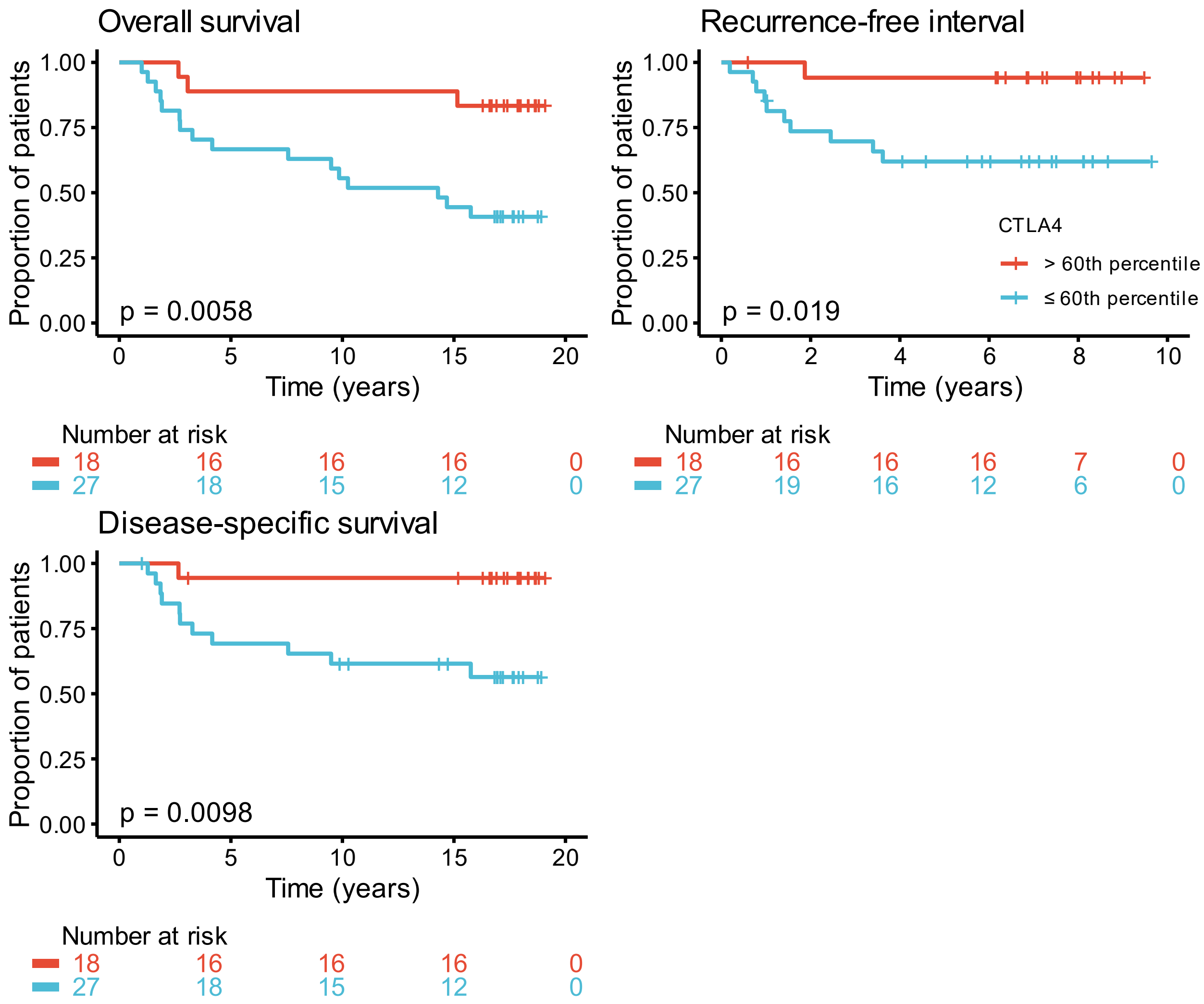

Supplementary Fig. 5 | Outcomes in Oslo1 by 60th percentile cutoff.

Overall survival, recurrence-free interval, and disease-specific survival in the Oslo1 cohort with *CTLA4* expression above and below the 60th percentile cutoff, derived from ROC analysis in the SCAN-B cohort.

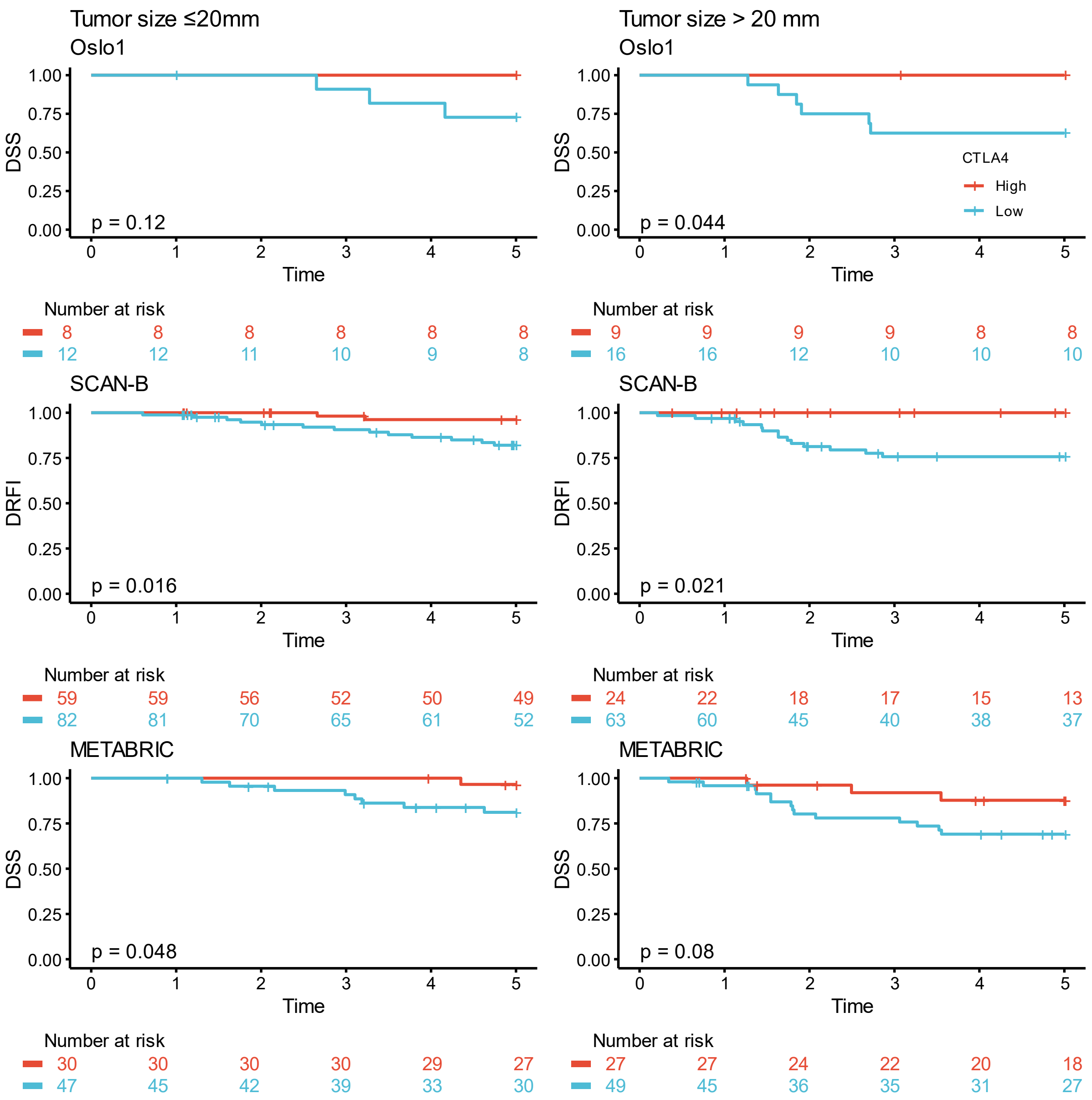

Supplementary Fig. 6 | Outcomes by *CTLA4* expression with tumors below and above 20 mm

Kaplan-Meier plots of disease-specific survival (DSS)/distant recurrence-free interval (DRFI) in patients with tumor size ≤20 mm (left panels) and >20 mm (right panels) in each cohort. This represents the size criterion for tumor stage T1 versus T2/T3. In each cohort, one patient had tumor size >50 mm (criterion for T3) and high *CTLA4* expression (> 63rd percentile); these patients were all event-free with observation times ranging from 4 to 5 years.

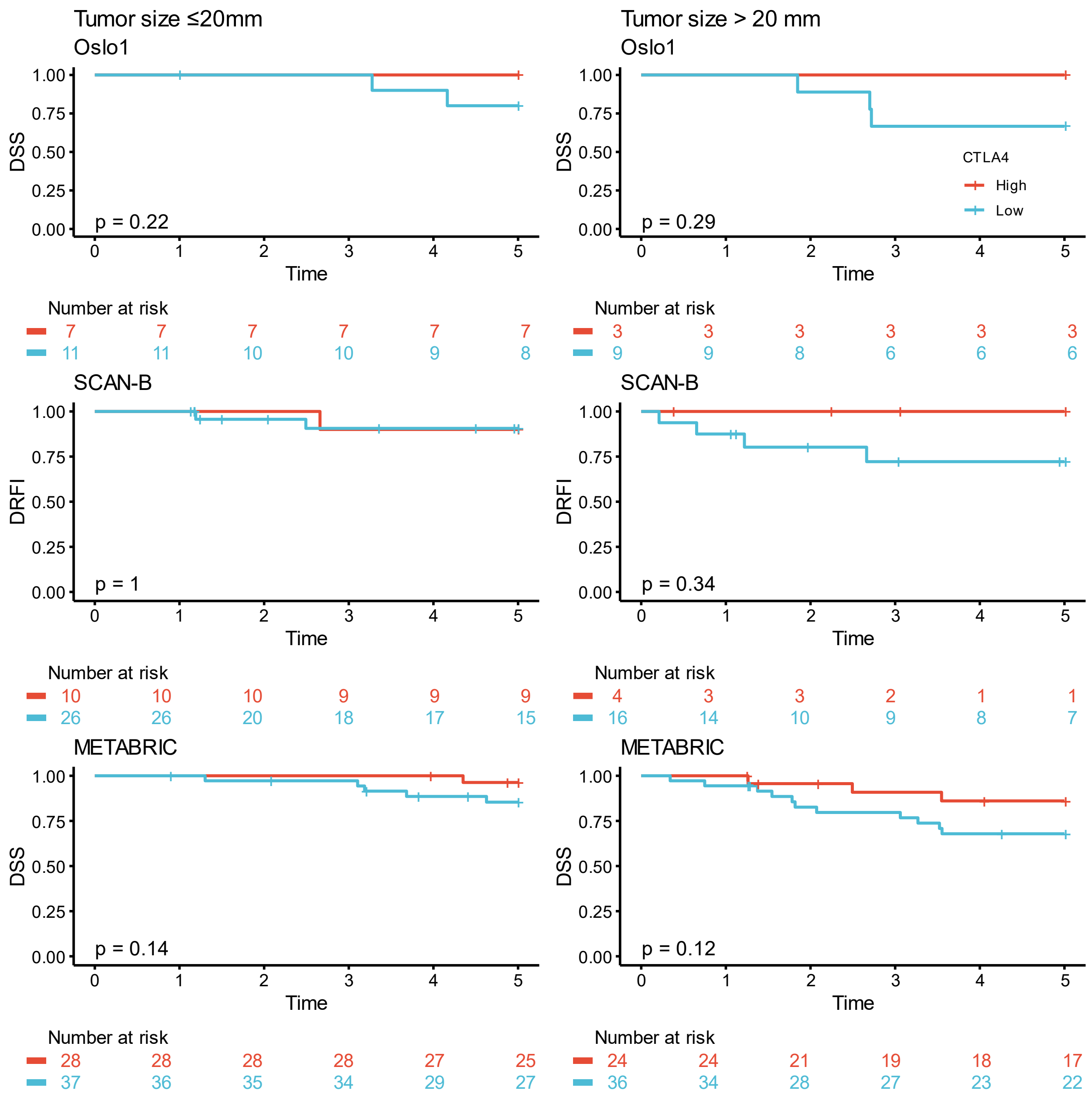

Supplementary Fig. 7 | Outcomes by *CTLA4* expression and tumor size in patients who did not receive adjuvant chemotherapy

Kaplan-Meier plots of disease-specific survival (DSS)/distant recurrence-free interval (DRFI) in patients with tumor size ≤20 mm (left panels) and >20 mm (right panels), who did not receive adjuvant chemotherapy. Only one patient, in the METABRIC cohort, had a tumor size >50 mm and high *CTLA4* expression (>63rd percentile). This patient was censored for disease-specific survival with an observation time of 4 years.

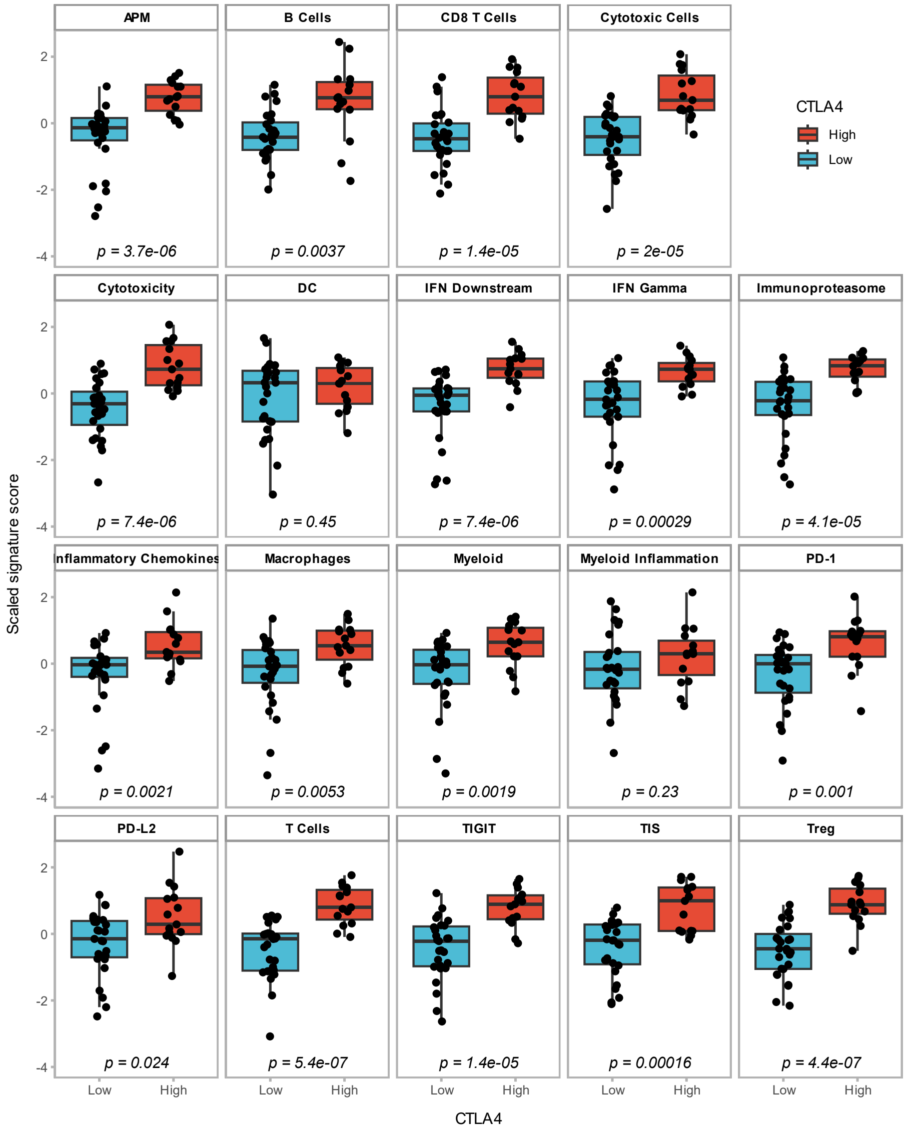

Supplementary Fig. 8 | *CTLA4* expression vs NanoString immune scores

CTLA4 expression above the 63rd percentile is considered high. Signature scores were computed by NanoString and have been scaled and centered at zero. Samples from all Oslo1 patients with available scores were included (n = 42). *P* values were calculated by the Wilcoxon rank sum method. Center lines represent median values, hinges the IQR, and whiskers the extreme values, omitting outliers extending >1.5 x IQR from the hinges.

APM, antigen processing machinery; DC, dendritic cells; IFN, interferon; TIS, tumor inflammation signature; Treg, regulatory T cells.

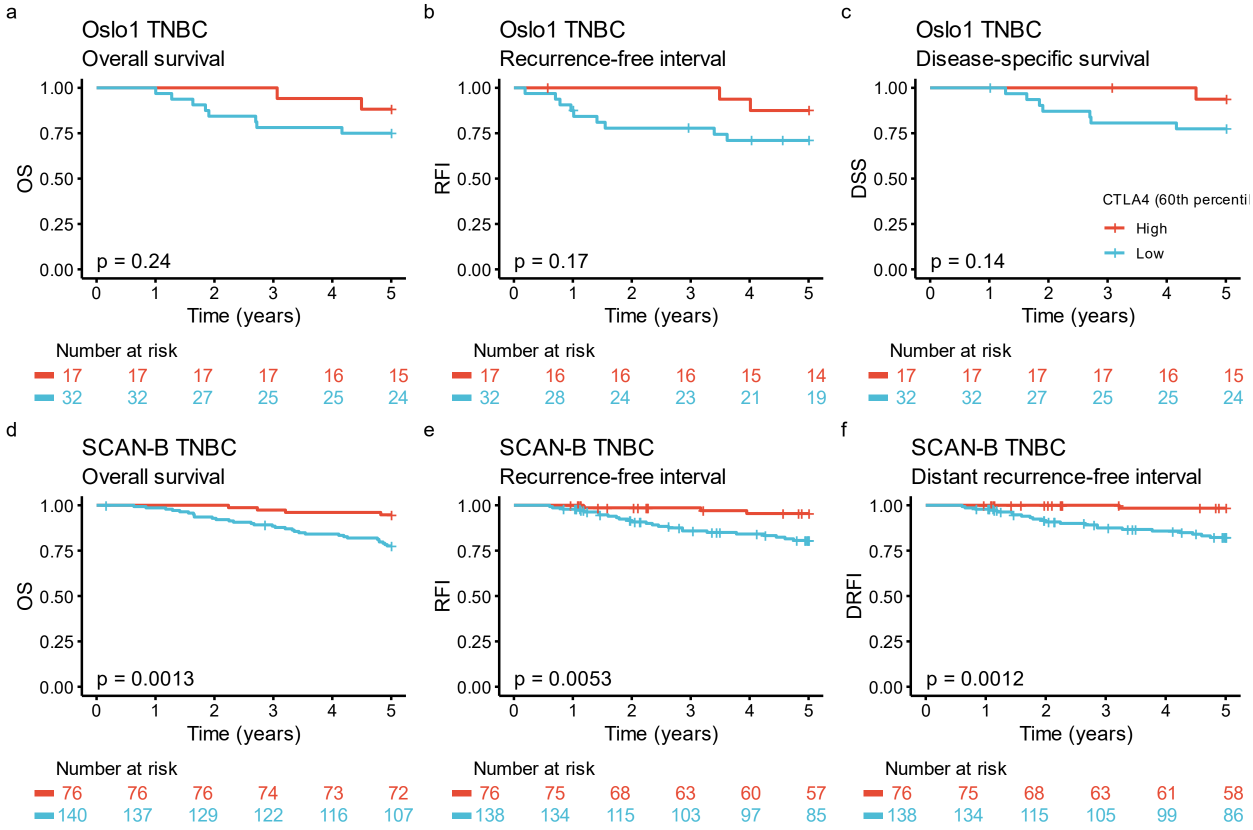

Supplementary Fig. 9 | Outcomes by *CTLA4* expression in triple-negative breast cancer, cutoff at 60th percentile

Survival/recurrence rates in patients with high and low *CTLA4* expression and triple-negative breast cancer (regardless of molecular subtype) in Oslo1 (a-c) and SCAN-B (d-f). For each cohort, the cutoff for high *CTLA4* expression was set at the 60th percentile of gene expression in basal-like samples in that cohort.

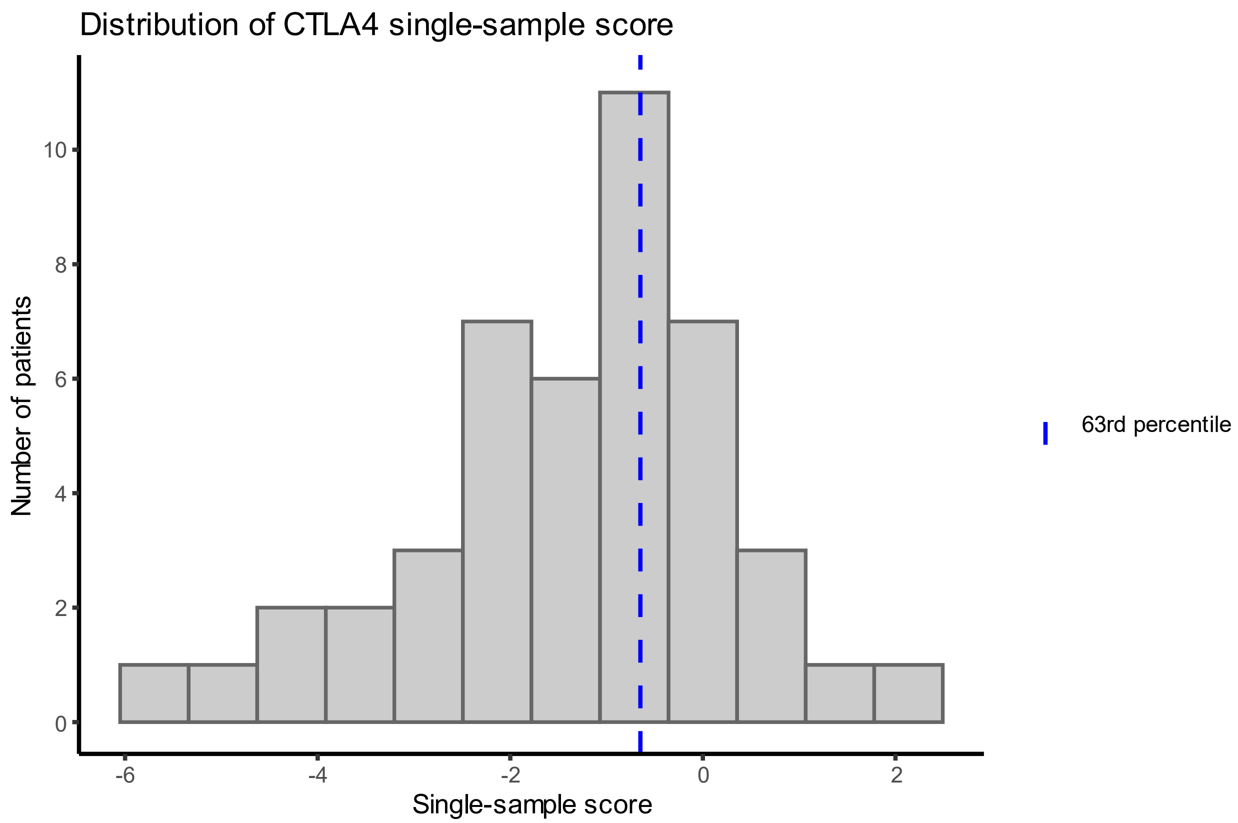

Supplementary Fig. 10 | Distribution of the single-sample *CTLA4* score.

Gene expression data from patients with basal-like node-negative disease in the Oslo1 cohort (n = 45) , analyzed on the NanoString PanCancer Immune Profiling Panel. The single-sample score represents the log_2-transformed ratio between *CTLA4* and 40 housekeeping genes. The dotted blue line indicates the optimized cutoff for predicting an excellent clinical outcome.

Oslo1	Univariable			Bivariable		
				CTLA4 controlled for predictor		
Predictors	*HR*	*95% CI*	*P*	*HR*	*95% CI*	*P*
Age	1.01	0.97 – 1.07	0.572	0.31	0.15 – 0.67	**0.003**
Size	0.84	0.47 – 1.50	0.549	0.42	0.24 – 0.75	**0.003**
Grade III	0.28	0.07 – 1.06	0.06	0.46	0.24 – 0.88	**0.019**
ER positive	0.95	0.12 – 7.46	0.964	0.4	0.22 – 0.73	**0.003**
TIL (10% increase)	0.74	0.51 – 1.09	0.131	0.46	0.23 – 0.90	**0.024**
ROR	0.98	0.95 – 1.01	0.196	0.42	0.22 – 0.80	**0.008**
CTLA4 (1 SD increase)	0.42	0.24 – 0.76	**0.004**			

Supplementary Table 1 | Uni- and bivariable Cox regression

Cox regression with disease-specific survival (DSS) as the dependent variable and *CTLA4* expression, age at diagnosis, tumor size, tumor grade, and ER status (≥10% positive cells by IHC) as the independent variables. Scaled *CTLA4* expression, age (years), and tumor size (cm) were included as continuous variables and TIL as a continuous variable in 10% increments, while tumor grade (grade I-II vs III) and ER status (positive vs negative) were included as categorical variables. Results of univariable regression with each predictor are shown on the left, and results for *CTLA4* controlled for each of the other predictors on the right.

CTLA4, cytotoxic T-lymphocyte-associated protein 4; ER, estrogen receptor; IHC, immunohistochemistry; TIL, tumor-infiltrating lymphocytes.

	Oslo1			SCAN-B			METABRIC		
	**Chemo (N=15)**	**No chemo (N=30)**	***P value***	**Chemo (N=174)**	**No chemo (N=57)**	***P value***	**Chemo (N=29)**	**No chemo (N=126)**	***P value***
**Age (years)**	**n = 15**	**n = 30**	***0.019***	**n = 174**	**n = 57**	***<0.001***	**n = 29**	**n = 126**	***0.002***
Mean (SD)	46.3 (10.1)	55.8 (13.0)		56.0 (12.4)	72.9 (13.8)		51.6 (9.46)	59.1 (12.2)	
**Tumor size (mm)**	**n = 15**	**n = 30**	***0.009***	**n = 170**	**n = 57**	*0.205*	**n = 28**	**n = 125**	*0.508*
Mean (SD)	29.5 (14.8)	21.2 (9.06)		20.2 (11.0)	20.4 (16.0)		26.9 (17.3)	24.0 (12.2)	
**Tumor grade**	**n = 15**	**n = 30**	*0.728*	**n = 162**	**n = 55**	***<0.001***	**n = 28**	**n = 124**	*0.177*
I	0	0		0	6 (10.5%)		0	3 (2.4%)	
II	2 (13.3%)	3 (10.0%)		5 (2.9%)	9 (15.8%)		1 (3.6%)	21 (16.9%)	
III	13 (86.7%)	27 (90.0%)		157 (90.2%)	40 (70.2%)		27 (96.4%)	100 (80.6%)	
**Histology**	**n = 15**	**n = 30**	*1*	**n = 173**	**n = 57**	***0.010***	**n = 29**	**n = 126**	*0.817*
IDC	13 (86.7%)	24 (80.0%)		154 (88.5%)	44 (77.2%)		28 (96.6%)	119 (94.4%)	
ILC	0	1 (3.3%)		1 (0.6%)	4 (7.0%)		0	4 (3.2%)	
Other	2 (13.3%)	5 (16.7%)		18 (10.3%)	9 (15.8%)		1 (3.4%)	3 (2.4%)	
**Receptor status**	**n = 15**	**n = 30**	*0.196*	**n = 164**	**n = 53**	***<0.001***			
TNBC	11 (73.3%)	26 (86.7%)		137 (78.7%)	37 (64.9%)				
HR+/HER2-	2 (13.3%)	3 (10.0%)		11 (6.3%)	16 (28.1%)				
HR-/HER2+	2 (13.3%)	0		11 (6.3%)	0				
HR+/HER2+	0	1 (3.3%)		5 (2.9%)	0				
**ER status**							**n = 28**	**n = 123**	*0.079*
Negative							26 (92.9%)	93 (75.6%)	
Positive							2 (7.1%)	30 (24.4%)	
**HER2 status**							**n = 18**	**n = 49**	*0.118*
Negative							13 (72.2%)	44 (89.8%)	
Positive							5 (27.8%)	5 (10.2%)	

Supplementary Table 2 | Clinicopathological variables in patients treated with and without chemotherapy

P values were calculated by the Wilcoxon rank-sum test for continuous variables and by Student's *t* test or Fisher's exact test (when appropriate) for categorical variables.

	CTLA4		
	**High (N=160)**	**Low (N=273)**	*P* value
**Age (years)**	**n = 160**	**n = 273**	*0.061*
Mean (SD)	56.7 (12.9)	59.6 (14.2)	
**Tumor size (mm)**	**n = 157**	**n = 270**	*0.034*
Mean (SD)	20.2 (10.4)	23.6 (14.4)	
**Tumor grade**	**n = 152**	**n = 264**	*0.007*
I	0	9 (3.3%)	
II	9 (5.6%)	32 (11.7%)	
III	143 (89.4%)	223 (81.7%)	
**Histology**	**n = 160**	**n = 272**	*0.032*
IDC	143 (89.4%)	241 (88.3%)	
ILC	0	10 (3.7%)	
Other	17 (10.6%)	21 (7.7%)	
**Receptor status**	**n = 95**	**n = 169**	*0.993*
TNBC	77 (81.1%)	136 (80.5%)	
HR+/HER2-	11 (11.6%)	21 (12.4%)	
HR-/HER2+	5 (5.3%)	8 (4.7%)	
HR+/HER2+	2 (2.1%)	4 (2.4%)	

Supplementary Table 3 | Distribution of other risk factors with high and low *CTLA4* basal-like BC

High *CTLA4* is defined as above the 66th percentile. *P* values are calculated by Wilcoxon's rank sum test for continuous variables and by the chi square test for categorical variables. All patients from the Oslo1, SCAN-B, and METABRIC cohorts with available data for each variable were included in the table.

	Oslo1			SCAN-B			METABRIC		
	***CTLA4* High (N=17)**	***CTLA4* Low (N=28)**	***P* value**	***CTLA4* High (N=86)**	***CTLA4* Low (N=147)**	***P* value**	***CTLA4* High (N=57)**	***CTLA4* Low (N=98)**	***P* value**
**Age (n)**	**n = 17**	**n = 28**	*0.051*	**n = 86**	**n = 147**	***0.020***	**n = 57**	**n = 98**	*0.396*
Mean (SD)	48.1 (9.53)	55.4 (13.9)		57.3 (13.6)	62.0 (15.1)		58.4 (11.6)	57.2 (12.3)	
**Tumor size (mm)**	**n = 17**	**n = 28**	*0.639*	**n = 83**	**n = 146**	***0.033***	**n = 57**	**n = 96**	*0.457*
Mean (SD)	22.2 (7.82)	25.0 (13.7)		17.8 (9.75)	22.1 (14.6)		23.0 (11.3)	25.4 (14.3)	
**Tumor grade**	**n = 17**	**n = 28**	*0.140*	**n = 78**	**n = 141**	*0.087*	**n = 57**	**n = 95**	*0.237*
I	0	0		0	6 (4.3%)		0	3 (3.2%)	
II	0	5 (17.9%)		3 (3.8%)	11 (7.8%)		6 (10.5%)	16 (16.8%)	
III	17 (100%)	23 (82.1%)		75 (96.2%)	124 (87.9%)		51 (89.5%)	76 (80.0%)	
**Histology**	**n = 17**	**n = 28**	*1*	**n = 86**	**n = 146**	*0.118*	**n = 57**	**n = 98**	*0.506*
IDC	14 (82.4%)	23 (82.1%)		73 (84.9%)	127 (87.0%)		56 (98.2%)	91 (92.9%)	
ILC	0 (0%)	1 (3.6%)		0 (0%)	5 (3.4%)		0 (0%)	4 (4.1%)	
Other	3 (17.6%)	4 (14.3%)		13 (15.1%)	14 (9.6%)		1 (1.8%)	3 (3.1%)	
**Receptor status**	**n = 17**	**n = 28**	*0.719*	**n = 78**	**n = 141**	*0.946*			
TNBC	13 (76.5%)	24 (85.7%)		64 (82.1%)	112 (79.4%)				
HR+/HER2-	2 (11.8%)	3 (10.7%)		9 (11.5%)	18 (12.8%)				
HR-/HER2+	1 (5.9%)	1 (3.6%)		4 (5.1%)	7 (5.0%)				
HR+/HER2+	1 (5.9%)	0 (0%)		1 (1.3%)	4 (2.8%)				
**ER status**							**n = 56**	**n = 95**	*0.329*
Negative							47 (83.9%)	72 (75.8%)	
Positive							9 (16.1%)	23 (24.2%)	
**HER2 status**							**n = 27**	**n = 40**	*0.509*
Negative							22 (81.5%)	35 (87.5%)	
Positive							5 (18.5%)	5 (12.5%)	

Supplementary Table 4 | Clinicopathological variables by *CTLA4* status in each cohort

P values were calculated by the Wilcoxon rank-sum test for continuous variables and by Student's *t* test or Fisher's exact test (when appropriate) for categorical variables.

SCAN-B	Univariable			Bivariable<br>*CTLA4* controlled for predictor		
Predictors	*HR*	*95% CI*	*P*	*HR*	*95% CI*	*P*
Age	1.01	0.98 – 1.03	0.676	0.56	0.39 – 0.80	**0.001**
Tumor size (cm)	1.36	1.13 – 1.65	**0.001**	0.63	0.45 – 0.90	**0.011**
Tumor grade III	0.8	0.24 – 2.62	0.707	0.55	0.38 – 0.80	**0.002**
ER positive	1.32	0.51 – 3.45	0.571	0.57	0.40 – 0.81	**0.002**
ROR	1	0.98 – 1.03	0.992	0.51	0.35 – 0.75	**0.001**
CTLA4 (1 SD increase)	0.57	0.40 – 0.80	**0.001**			

METABRIC	Univariable			Bivariable<br>*CTLA4* controlled for predictor		
Predictors	*HR*	*95% CI*	*P*	*HR*	*95% CI*	*P*
Age	0.98	0.95 – 1.00	0.088	0.67	0.46 – 0.98	**0.037**
Size (cm)	1.33	1.08 – 1.64	**0.008**	0.72	0.50 – 1.05	0.09
Grade III	1.15	0.48 – 2.77	0.758	0.65	0.44 – 0.96	**0.031**
ER positive	0.95	0.41 – 2.18	0.904	0.66	0.45 – 0.96	**0.031**
CTLA4 (1 SD increase)	0.7	0.48 – 1.00	0.053			

Supplementary Table 5 | Uni- and bivariable Cox regression models in the validation cohorts

Distant recurrence-free survival (DRFI) was used as the outcome measure in SCAN-B, and disease-specific survival (DSS) in METABRIC.

CTLA4 expression, age at diagnosis, tumor size, tumor grade, and ER status were used as independent variables. Scaled *CTLA4* expression, age (years), and tumor size (cm) were included as continuous variables and TIL as a continuous variable in 10% increments, while tumor grade (grade I-II vs III) and ER status (positive vs negative) were included as categorical variables. Results of univariable regression with each predictor are shown on the left, and results for *CTLA4* controlled for each of the other predictors on the right.
