# Supplementary material for: High tumor expression of CTLA4 identifies lymph node-negative basal-like breast cancer patients with excellent prognosis
Source: Commun Med (Lond). 2025 Jun 16;5:234. doi: 10.1038/s43856-025-00865-z (PMC12170890; doi:10.1038/s43856-025-00865-z)
Supplement: Supplementary file 2 — Description of Additional Supplementary Materials [file 43856_2025_865_MOESM2_ESM.pdf]

Description of Additional Supplementary Files

File name: Supplementary Data 1

Description: Source data underlying the analyses
